# Fecal microbiota from MRL/lpr mice exacerbates pristane-induced lupus

**DOI:** 10.1186/s13075-023-03022-w

**Published:** 2023-03-16

**Authors:** Xiaoqing Yi, Cancan Huang, Chuyi Huang, Ming Zhao, Qianjin Lu

**Affiliations:** 1 Department of Dermatology, Second Xiangya Hospital, Central South University, Changsha, 410011 China; 2 Research Unit of Key Technologies of Diagnosis and Treatment for Immune-related Skin Diseases, Chinese Academy of Medical Sciences, Changsha, 410011 China; 3 Clinical Medical Research Center of Major Skin Diseases and Skin Health of Hunan Province, Changsha, 410011 China; 4 Hunan Key Laboratory of Medical Epigenomics, Second Xiangya Hospital, , Central South University, Changsha, 410011 China; 5 Institute of Dermatology, Chinese Academy of Medical Sciences and Peking Union Medical College, Nanjing, China; 6 Key Laboratory of Basic and Translational Research on Immune-Mediated Skin Diseases, Chinese Academy of Medical Sciences, Nanjing, China; 7 Jiangsu Key Laboratory of Molecular Biology for Skin Diseases and STIs, Nanjing, China

**Keywords:** SLE, Metagenomic sequencing, Metabolomics, Prevotella, Valine, L-Isoleucine, Microbiota, MRL/lpr

## Abstract

**Background:**

The roles of gut microbiota in the pathogenesis of SLE have been receiving much attention during recent years. However, it remains unknown how fecal microbiota transplantation (FMT) and microbial metabolites affect immune responses and lupus progression.

**Methods:**

We transferred fecal microbiota from MRL/lpr (Lpr) mice and MRL/Mpj (Mpj) mice or PBS to pristane-induced lupus mice and observed disease development. We also screened gut microbiota and metabolite spectrums of pristane-induced lupus mice with FMT via 16S rRNA sequencing, metagenomic sequencing, and metabolomics, followed by correlation analysis.

**Results:**

FMT from MRL/lpr mice promoted the pathogenesis of pristane-induced lupus and affected immune cell profiles in the intestine, particularly the plasma cells. The structure and composition of microbial communities in the gut of the FMT-Lpr mice were different from those of the FMT-Mpj mice and FMT-PBS mice. The abundances of specific microbes such as prevotella taxa were predominantly elevated in the gut microbiome of the FMT-Lpr mice, which were positively associated with functional pathways such as cyanoamino acid metabolism. Differential metabolites such as valine and L-isoleucine were identified with varied abundances among the three groups. The abundance alterations of the prevotella taxa may affect the phenotypic changes such as proteinuria levels in the pristane-induced lupus mice.

**Conclusion:**

These findings further confirm that gut microbiota play an important role in the pathogenesis of lupus. Thus, altering the gut microbiome may provide a novel way to treat lupus.

**Supplementary Information:**

The online version contains supplementary material available at 10.1186/s13075-023-03022-w.

## Introduction

Systemic lupus erythematosus (SLE) is a complex autoimmune disease with multiple clinical manifestations caused by various aetiologies involving genetic, hormonal, and environmental factors [1]. Recently, much attention has been given to the roles of the microbiome in the initiation, development, exacerbation, or remission of SLE in patients, whether from the aspects of the skin, gut, or oral cavity [2–4]. With the advance of culture-independent multiomics sequencing, we are able to gain deep and comprehensive insights into microbial communities and have established specific links between several candidate microbial taxa and SLE pathogenesis. For example, *Staphylococcus* species, especially *Staphylococcus aureus* and *Staphylococcus epidermis*, are enriched and colonize primarily in the skin lesions of SLE and may be potentially reliable noninvasive biomarkers for SLE [3]. High levels of serum antibodies restricted to *Ruminococcus gnavus* have potential prognostic value for the risk of lupus nephritis [5]. The increased gut colonization of *Lactobacillus* repairs the destroyed intestinal barriers and attenuates renal damage by suppressing proinflammatory factors [6]. Although much effort has been made to explore the causal role of specific pathogens in inflammation and autoimmunity, the roles and mechanisms that the gut microbes play in the pathogenesis of SLE remain unclear.

The MRL/lpr mouse model is a kind of spontaneous murine model prone to producing lupus-like phenotypes and can meet the genetic requirements for SLE induction [7]. The MRL/lpr mice displayed high concentrations of circulating autoantibodies, resulting in the formation of large amounts of immune complexes and severe nephritis [8], which was attributed to the deficiency of fas signalling, thereby promoting autoimmunity in MRL/lpr mice [9, 10]. The MRL/Mpj mice, as the control strain of MRL/lpr mice, have a delayed onset of autoimmune disorders and exhibit milder glomerulonephritis despite carrying the normal fas gene [11, 12]. Therefore, MRL/Mpj mice still have a potential risk of lupus. Previous researchers have found differences in the gut bacterial composition and diversity between MRL/lpr mice and MRL/Mpj mice, and the gut microbiota dynamically changes over time during disease progression [13], which means that the gut microbiota may play a crucial role in the disease development of the MRL mouse strain.

Pristane-induced lupus mice are a kind of mouse model extensively employed in scientific research examining the pathophysiology of SLE, as these mice can exhibit mild glomerulonephritis and the production of several autoantibodies, including anti-dsDNA antibodies, mimicking the clinical features of SLE patients [14, 15]. Although this mouse model is not genetically susceptible to developing lupus, the induction pathways in this model are similar to SLE pathogenesis, such as the overproduction of IFN-γ. Therefore, the pristane-induced lupus model tends to be used for investigating the environmental triggers in disease development [16]. However, to date, the microbial factors in the pristane-induced lupus mice are still poorly understood.

In this study, fecal microbiota from MRL/lpr mice and MRL/Mpj mice were transferred to pristane-induced lupus mice to investigate the role of the gut microbiota cross-interaction and metabolite reciprocity between spontaneous and induced murine models in lupus development. We performed a comprehensive metagenomic and metabolomic analysis of the gut microbial communities in pristane-induced lupus mice with FMT. This study will provide clues for demonstrating the role of the gut microbiota in the pathogenesis of SLE and developing a novel strategy for SLE therapy.

## Materials and methods

### Pristane-induced lupus mouse model

Female C57/BL mice aged 8 weeks were injected with 500 μl pristane intraperitoneally (Sigma, P9622) and maintained under specific-pathogen-free environment. The proteinuria levels were measured every week using the colorimetric assay strip (URIT, China) according to the scale for assessment: 0 = absent; ± = 15 mg dl − 1; + = 30 mg dl − 1; + + = 100 mg dl − 1; + + + = 300 mg dl − 1; and + + + + ≥ 500 mg dl − 1. At the 9th month, the end of the experiment, mice were anesthetized and the serum samples were collected by cardiac puncture and the anti-dsDNA IgG levels were assessed by ELISA (Alpha Diagnostic,5120).

### Fecal microbiota transplantation and fecal sample collection

All the mice were orally gavaged with the antibiotic mixture consisting of ampicillin (6.7 mg/ml), neomycin (6.7 mg/ml), vancomycin (3.35 mg/ml), and metronidazole (6.7 mg/ml) dissolved in the sterile water, 250 µl per mouse for 7 days for the gut purge [17], followed with 7 days of resting, then started with the fecal microbiota transplantation or were orally gavaged with PBS as the control group, three times a week. Fecal pellets were freshly prepared and sampled from the MRL/lpr mice (15–18 weeks age, proteinuria > = + + +) or MRL/Mpj mice (15–18 weeks age), placed on the ice temporarily. Then the collected fecal materials were pooled and suspended with sterile PBS by the vortex with the ratio of 100 mg feces: 1 ml PBS. The fecal suspension was filtered through the 100-μm sieve, and the filtered fecal microbiota was introduced to the recipient mice at 0.2 ml/mice [6].

Fecal samples were collected for the subsequent microbiota and metabolite sequencing at three time points: the baseline time before the fecal microbiota transplantation, the middle time (at the 5th month), the final time at the end of the experiment. Fecal pellets of each mouse were stored in the sterile EP tubes, quickly frozen in liquid nitrogen and then transferred to the freezer awaiting further extraction and analysis.

Fecal microbiota from MRL/lpr mice, MRL/Mpj mice, or PBS transferred to the pristane-induced lupus-like mouse were termed as FMT-Lpr, FMT-Mpj, and FMT-PBS. The sample size of each group is 11 for FMT-Lpr, 10 for FMT-Mpj, and 7 for FMT-PBS.

### Histology

Renal tissues and intestinal tissues (sampled at the ileum and colon) were fixed with paraformaldehyde and embedded with paraffin. The sections were stained with H&E for observing the histological features. The renal tissues were also stained with recombinant anti-C3 antibody [EPR19394] (abcam, ab200999) or goat anti-mouse IgG antibody conjugated with Alexa Fluor 488 (abcam, ab150113) by immunofluorescence for observing the immune complex depositions in the kidney. The intestinal tissues were stained with the anti-ZO1 tight junction antibody (abcam, ab96587).

### Quantitative RT-PCR

Extracted RNA from renal and intestinal tissues using trizol reagent was further reversely transcripted into cDNA and analyzed the relative expression levels of several genes with a LightCycler 96 System (Roche, Switzerland). Primer sequences were obtained as required from Tsingke Biotechnology Co, Ltd and listed in the Supplementary Table 1. The relative expression levels of target genes were calculated by the 2^−ΔΔCT^ method, normalized to the reference gene Actb.

### Flow cytometry

Single-cell suspensions from the spleen and lymph nodes were prepared and fluorescently labeled as previously described [18]. The Leukocyte Activation Cocktail with BD GolgiPlug™ antibody (BD, 550,583) and Cytofix/CytopermTM Fixation/Permeabilization Kit (BD, 554,714) were purchased for the intracellular cytokine detection. The Foxp3/Transcription Factor Fixation/Permeabilization Concentrate and Diluent (eBioscience, 00–5521-00) was used for Gata3 staining.

The renal and intestinal tissues were cut into pieces and digested in the solution containing collagenase IV (1 mg/ml) (sigma, V900893), DNase I (100 μg/mL) (sigma, DN25), and dispase (1 mg/mL) (sigma, 04,942,078,001) for 1 h. Then, the solution was passed through a cell strainer (aperture size: 70 μm) and the leukocytes were further separated with 35% percoll and washed with PBS twice for the following flow cytometry.

The flowing antibodies were used: APC-Cy™7 hamster anti-mouse CD3e, FITC rat anti-mouse CD8a (BD, 557,596), PE-Cy™7 rat anti-mouse CD4 (BD, 552,775), APC rat anti-mouse IFN-γ (BD, 554,413), PE rat anti-mouse IL-4(BD, 554,435), PerCP-Cy™5.5 rat anti-mouse IL-17A (BD, 560,666), FITC anti-mouse B220 (Biolegend, 103,205), PE rat anti-mouse IgD (Biolegend, 558,597), BV421 rat anti-mouse CD138 (Biolegend, 562,610), Zombie NIR™ Fixable Viability Kit (Biolegend, 423,105), APC/Cyanine7 anti-mouse CD45 (Biolegend 103,116), Alexa Fluor® 700 anti-mouse Ly-6G (Biolegend, 127,622), Alexa Fluor® 647 anti-mouse/human CD11b (Biolegend, 101,218), PE/Cy7 anti-mouse CD335 (NKp46) (Biolegend, 137,618), PerCP/Cyanine5.5 anti-mouse Ly-6C (Biolgend, 128,012), PE/Cy5 anti-mouse CD3ε (Biolegend, 100,310), PE Mouse anti-Mouse RORγt (BD, 562,607), Alexa Fluor® 488 Hamster Anti-Mouse KLRG1 (BD, 561,619), BB515 Rat Anti-Mouse CD19 (BD,550,992), Alexa Fluor® 700 anti-mouse CD8a (Biolegnd, 100,730), PE/Cy7 anti-mouse CD62L (Biolegend, 104,418), PerCP/Cyanine5.5 anti-mouse/human CD45R/B220 (Biolegend, 103,236), PE/Dazzle™ 594 anti-mouse/human CD127 (Biolegend, 101,256), PE anti-mouse/human CD44 (Biolegend, 103,007), Alexa Fluor® 488 anti-mouse IgD (Biolegend, 405,718), BB700 rat anti-mouse CD4 (BD, 566,407), TruStain fcX (Biolegend, 101,320), Fixable Viability Stain 510 (BD, 564,406). All samples were detected by Flow Cytometry DxP AthenaTM (Cytek). The flow gating strategies for cells isolated from the spleen, lymph nodes, kidney, and intestines are shown in supplementary Fig. 1.

### 16 s rRNA sequencing

The extracted DNA from fecal samples was acquired according to the E.Z.N.A.® soil DNA Kit’s instructions (Omega Bio-tek, USA). The bacterial 16S rRNA were amplified at the hypervariable V3–V4 region with primer pairs (forward primer: 5′-ACTCCTACGGGAGGCAGCAG-3′ and reverse primer: 5′-GGACTACHVGGGT.

WTCTAAT-3′) by PCR according to a step cycling protocol. Then, the PCR products were extracted, purified, and quantified. The purified amplicons were pooled, paired-end, and further sequenced on an Illumina MiSeq PE300 platform (Illumina, USA). The raw sequence reads were processed by fastp [19] and merged by FLASH [20]. The OTU was picked by RDP Classifier [21] and further analysis such as alpha diversity was performed.

### Metagenomic sequencing

Metagenomic sequencing was prepared and conducted on the platform of Shanghai Majorbio company as previously described [22]. Briefly, DNA was firstly extracted, and fragmented for library construction, then started sequencing on Illumina NovaSeq (Illumina Inc, USA) and performed sequence quality control and genome assembly. Next, open reading frames (ORFs) in final-assmbling contigs were predicted using MetaGene [23] for gene prediction and non-redundant gene catalog was constructed for taxonomy and functional annotation. More detailed informative analysis was implemented as required.

### Metabolomics

Fecal metabolites of each sample underwent the process of quality control and performed the Chromatographic separation on a Thermo UHPLC system equipped with an ACQUITY BEH C18 column (Waters, Milford, USA). After the UPLC-TOF/MS Thermo UHPLC-Q Exactive Mass Spectrometer analyses, data were preprocessed, normalized, and imputed. Mass spectra of these metabolic features were identified and annotated in reliable biochemical databases such as metlin or HMDB database. Data were log-transformed for the following statistical analysis such as multivariate statistical analysis and differential metabolite analysis.

### Statistical analysis

All data were exhibited in the form of mean ± SD and performed the statistical analyses in the GraphPad prism. The statistical significance was determined by one-way ANOVA and post hoc comparison. Anderson–Darling test was used for testing the normal distribution. Pearson correlation coefficients were calculated for analyzing the strength of the correlation for normally distributed data; otherwise, Spearman correlation coefficients were employed. The *p* value less than the threshold of 0.05 was regarded as a significant difference.

## Results

### Fecal microbiota from MRL/lpr mice aggravated lupus autoimmunity

**Fig. 1:**
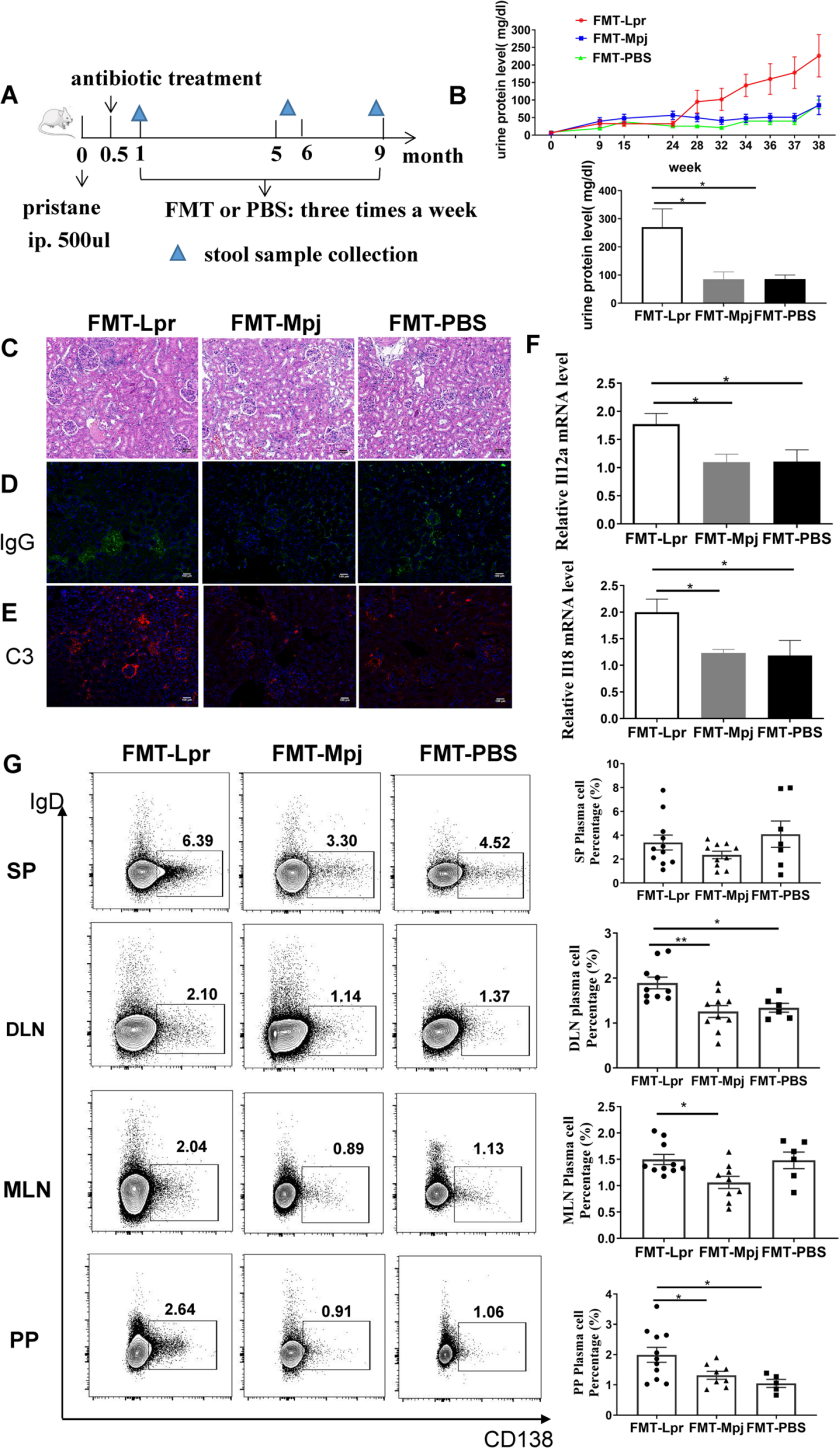
FMT-Lpr mice accelerated the lupus pathogenesis. **A** The schematic timeline of the experiment design. **B** Dynamic changes of urine protein levels in FMT-Lpr mice, FMT-Mpj mice, and FMT-PBS mice with the disease progression (upper) and urine protein levels measured at the final week of the experiment period (down). **C** Representative images of HE staining of kidney sections. Representative images of IgG (**D**) and C3 (**E**) depositions in the kidney detected by immunofluorescence. Scale bar, 100 μm. magnification × 400. **F** Il12a, Il18 mRNA expression levels in the kidney. **G** Flow cytometric analysis of the plasma cell percentages gated on the CD45 + cells in the spleens and lymph nodes (SP: spleen; DLN: draining lymph node; MLN: mesentery lymph nodes; PP: peyer’s patch). *p < 0.05. FMT-Lpr, *n* = 10 ~ 11; FMT-Mpj, *n* = 9 ~ 10; FMT-PBS, *n* = 6 ~ 7

To investigate the effects of FMT on the development of lupus disease, we transferred the fecal microbiota of MRL/lpr mice, that of MRL/Mpj mice or PBS gavage to the pristane-induced lupus-like mouse model (respectively termed as FMT-Lpr, FMT-Mpj, and FMT-PBS) (Fig. 1A). In the final observation stage, the urine protein levels of the FMT-Lpr mice were significantly elevated compared to those of the FMT-Mpj mice and FMT-PBS mice (*p*_FMT-Lpr VS FMT-Mpj_ = 0.0142; *p*_FMT-Lpr VS FMT-PBS_ = 0.0349) (Fig. 1B). Hematoxylin and eosin (H&E) staining and immunofluorescence of the renal sections revealed more severe glomerular abnormalities (Fig. 1C**)** as well as more IgG and C3 depositions in the glomeruli of the FMT-Lpr mice (Fig. 1D,E). We further detected the mRNA expression levels of proinflammatory cytokines in the kidney and found significantly increased Il12a and Il18 mRNA expression in FMT-Lpr mice compared to FMT-Mpj mice and PBS-gavaged mice (Il12a: *p*_FMT-Lpr VS FMT-Mpj_ = 0.0112; *p*_FMT-Lpr VS FMT-PBS_ = 0.0427. l118: *p*_FMT-Lpr VS FMT-Mpj_ = 0.0111; *p*_FMT-Lpr VS FMT-PBS_ = 0.0484) (Fig. 1F, Figure S2A). The percentages of T and B lymphocytes in the renal tissues were not significantly different among the three groups (Figure S2B).

Fecal microbiota transplantation had no obvious effect on the production of the anti-dsDNA antibodies (Figure S2C). The percentages of Th1, Th2, and Th17 cells in the spleen and lymph nodes were not significantly different among the three groups (Figure S2D). The increased percentages of plasma cells could be identified in the lymph nodes rather than the spleens of the FMT-Lpr mice, including draining lymph nodes, mesentery lymph nodes, and Peyer’s patches. (DLN: *p*_FMT-Lpr VS FMT-Mpj_ = 0.0033; *p*_FMT-Lpr VS FMT-PBS_ = 0.0274. MLN: *p*_FMT-Lpr VS FMT-Mpj_ = 0.0193. PP: *p*_FMT-Lpr VS FMT-Mpj_ = 0.0077; *p*_FMT-Lpr VS FMT-PBS_ = 0.0108) (Fig. 1G). Therefore, the transplantation of fecal microbiota from MRL/lpr mice exacerbates the renal damage of the pristane-induced lupus mouse model.

### FMT changed the intestinal immunological states in pristane-induced lupus mice

**Fig. 2:**
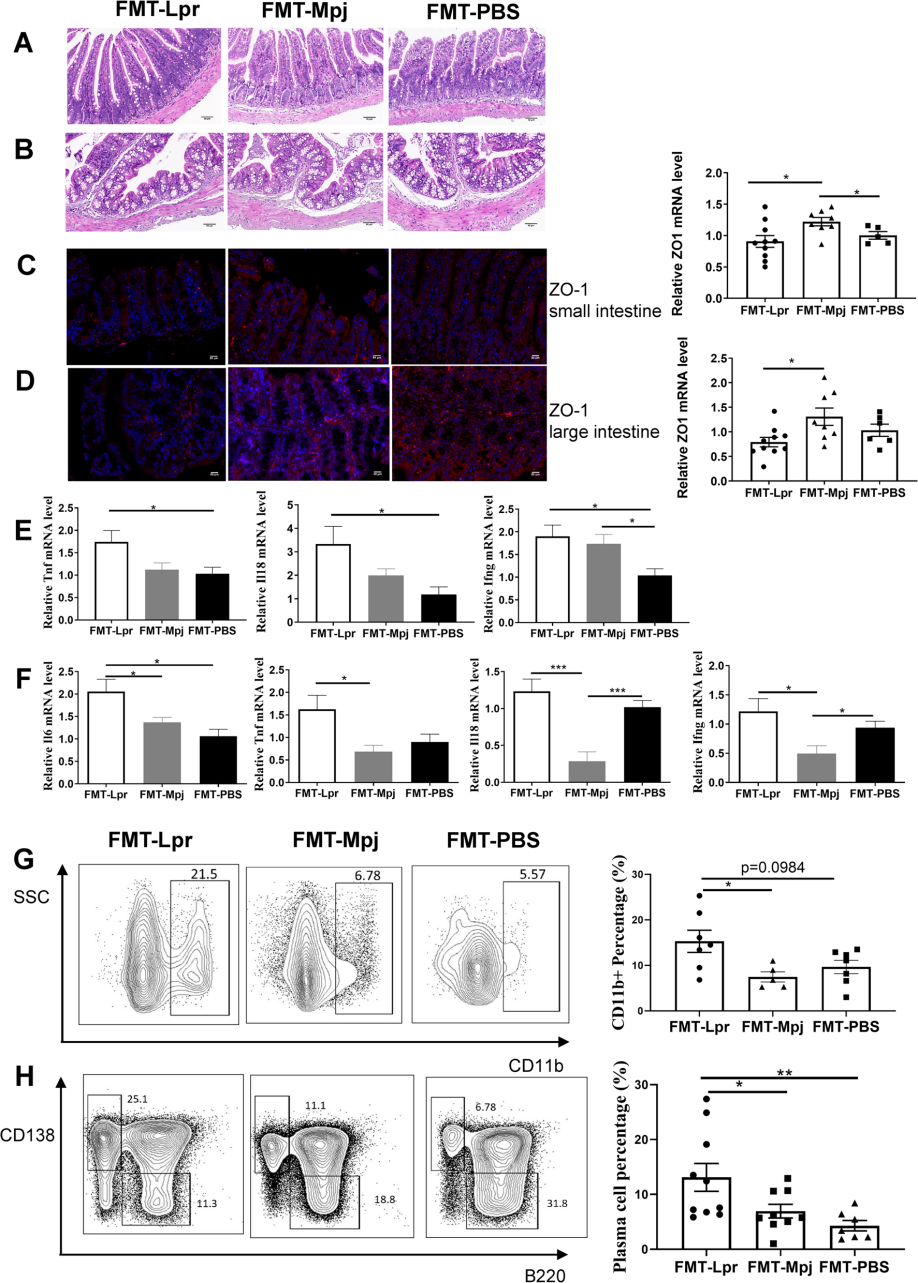
FMT-Lpr mice have altered intestinal permeabilities and immunological states. Representative images of HE staining of the small intestine (**A**) and the large intestine (**B**). Scale bar, 50 μm. magnification × 400. ZO-1 expressions detected by immunofluorescence and the mRNA levels measured by RT-PCR in the small intestine (**C**) and the large intestine (**D**). Tnf, Il18, and Ifng mRNA expression levels in the small intestine (**E**) and Il6, Tnf, Il18, and Ifng mRNA expression levels in the large intestine (**F**). Flow cytometric analysis of the percentages of CD11b + cells gated on the CD45 + CD3 − CD19 − cells in the small intestine (**G**) and the percentages of B220 − IgD − CD138 + cell characterized as the plasma cells gated on the CD45 + cells in the large intestine (**H**). *p < 0.05. **p < 0.001. FMT-Lpr, *n* = 8 ~ 11; FMT-Mpj, *n* = 5 ~ 10; FMT-PBS, *n* = 5 ~ 7

Pristane-induced lupus mice gavaged with fecal microbiota, regardless of the donor source being MRL/lpr mice or MRL/Mpj mice, maintained intestinal structural integrity without obvious inflammatory infiltration in the small or large intestine, which was identified by H&E staining (Fig. 2A,B). To test whether the intestinal permeability was affected during the fecal microbiota transplantation, we measured the transcription level of ZO-1, a tight junction protein and found the decreased mRNA expression of ZO-1 in the small and large intestines of the FMT-Lpr mice (small intestine: *p*_FMT-Lpr VS FMT-Mpj_ = 0.0190; *p*_FMT-Mpj VS FMT-PBS_ = 0.0463. large intestine: *p*_FMT-Lpr VS FMT-Mpj_ = 0.0243), which was further validated by the immunofluorescence (Fig. 2C,D). Therefore, FMT from MRL/lpr mice may affect intestinal barrier function. We also detected the transcript levels of proinflammatory cytokines and revealed that the mRNA expression of Tnf, Il18, Ifng, and Il6 was significantly elevated in the small or large intestines of FMT-Lpr mice compared with FMT-Mpj mice or FMT-PBS mice (Fig. 2E,F, Figure S3A-B).

Furthermore, to investigate the composition of immune cells in the intestinal mucosa, we performed flow cytometry to analyze the percentages of T cells, B cells, innate lymphoid cells (ILCs), and CD11b + cell subsets (Figure S1C). The proportions of ILC1s and ILC3s were not significantly different among the three groups. However, the ILC2 proportion was increased in the FMT-Lpr mice and FMT-Mpj mice compared with FMT-PBS mice in the large intestine (Figures S3C-D). Furthermore, in both FMT-Lpr mice and FMT-Mpj mice, the CD8 + T-cell percentages were decreased in the small intestine, and the CD4 + T-cell percentages were reduced in the large intestine compared with those of FMT-PBS mice (Figure S3E-F), indicating that the altered tendencies of T lymphocytes are FMT-related instead of lupus-related. CD4 + and CD8 + T cells in both sites were dominated by the effector subtype characterized by increased proportions of the CD44 + CD62L − subset (Figure S3G-H). The CD11b + cell percentage in the large intestine was also associated with FMT (Figure S3I). We further subclassified CD11b + cells based on the Ly6C and Ly6G markers, of which the Ly6C-Ly6G + subset was considered Ly6G + neutrophils and the Ly6C + Ly6G − subset was representative of Ly6C + monocytes/macrophages. The percentage of Ly6G + neutrophils was significantly increased in the large intestine of the FMT-Lpr mice and FMT-Mpj mice compared with the FMT-PBS mice, while the percentage of Ly6C + monocytes/macrophages showed no significant differences among the three groups (Figure S2J). Remarkably, FMT from MRL/lpr mice but not MRL/Mpj mice increased the CD11b + cell percentage in the small intestine (*p*_FMT-Lpr VS FMT-Mpj_ = 0.0318) (Fig. 2G) as well as the plasma cell percentage in the large intestine (*p*_FMT-Lpr VS FMT-Mpj_ = 0.0311; *p*_FMT-Mpj VS FMT-PBS_ = 0.0098) (Fig. 2H). Therefore, FMT can affect the intestinal immunological status of pristane-induced lupus mice. Feces from MRL/lpr mice could alter specific immune cell types, such as plasma cells in the intestine. These findings also indicated that fecal microbiota from different origins has differentiated pathological and immunological effects.

### FMT altered the gut microbial structure and composition in the pristane-induced lupus mice

**Fig. 3:**
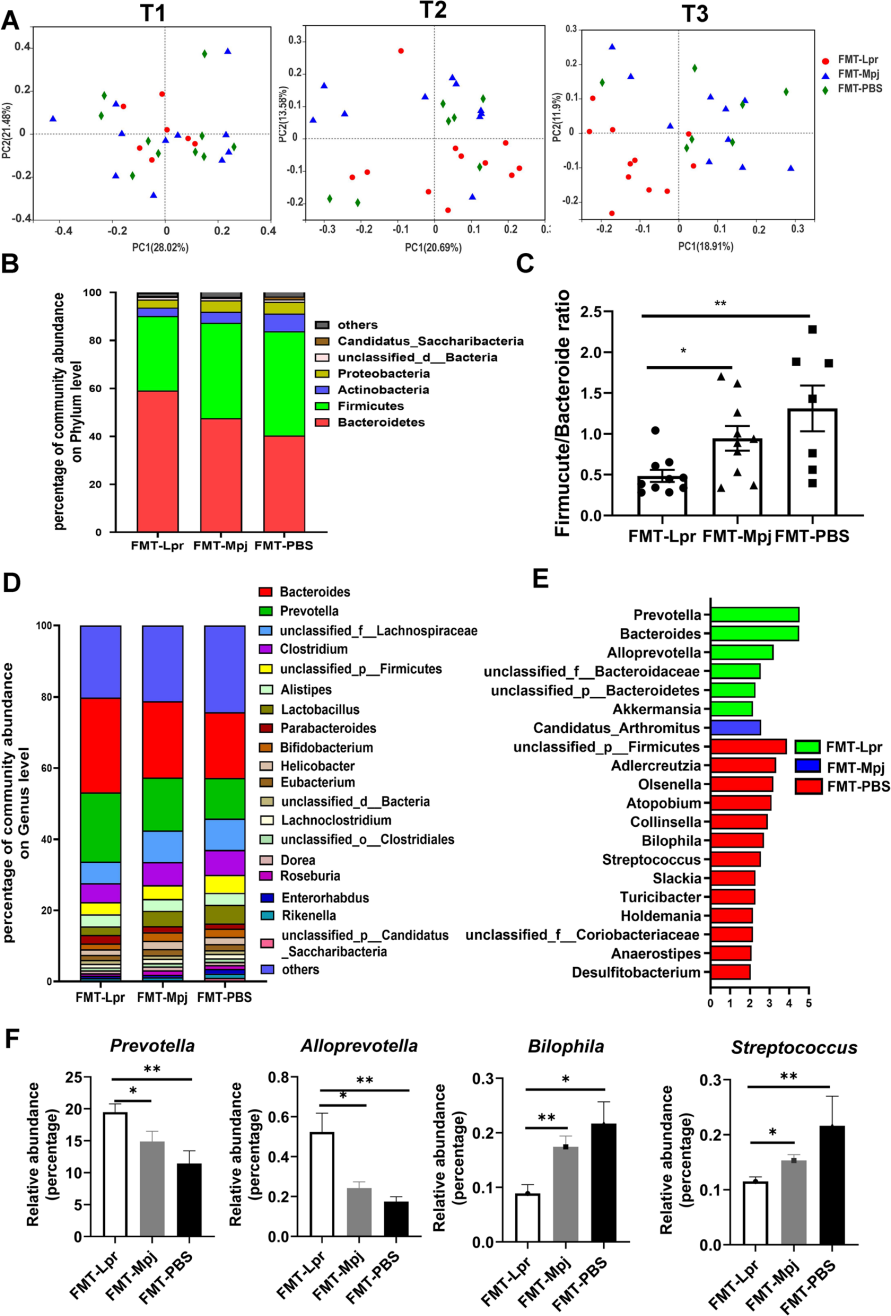
FMT changed the microbial community structures and compositions. **A** The sample distribution of the gut microbiome in the principal coordinate analysis (PCoA) plot at different time points. Different color represents different group (red: FMT-Lpr, blue: FMT-Mpj, green: FMT-PBS). T1, T2, and T3 respectively refer to the beginning, middle and end of the experiment. **B** Profiles of the major phylum with the relative abundance > 0.01 per group. **C** The Firmicute/Bacteroide ratio among the three groups. **D** Profiles of the relative abundances of the top 20 predominant taxa per group at the genus level. **E** Characteristic bacterial taxa for each group shown by LDA score > 2.0 at the genus level. **F** The relative abundances of the *prevotella*, *alloprevotella*, *streptococcus*, and* bilophila* among the three groups. *p < 0.05. **p < 0.001. FMT-Lpr, *n* = 11; FMT-Mpj, *n* = 10; FMT-PBS, *n* = 7

We next investigated whether the gut microbial structure and composition were associated with FMT and disease progression. Fecal samples from the three groups were collected at the beginning (the first week after the intraperitoneal injection of pristane, termed T1), middle (the 5th month, termed T2), and end of the experiment (the 9th month, at the end of the experiment, termed T3), and 16S rRNA sequencing was performed to clarify the most significant discrepancy in the gut microbial structure at different stages of the lupus process. According to the 16S rRNA sequencing results, we found no distinct differences in alpha diversities among the three groups in terms of richness, evenness, and overall diversity at each time point of sample collection, as indicators such as the Shannon, Simpson, and Chao indices were compared among the three groups (Table S2). However, during the progression of lupus, the gut bacterial community of FMT-Lpr mice became gradually distinct from that of FMT-Mpj mice and FMT-PBS mice, especially at the end of the experiment, consistent with the phenotypic changing trends (Fig. 3A). Moreover, the gut microbiome could be optimally partitioned into two distinct clusters based on the structure of advantageous flora at the phylum level, as indicated by the Calinski‒Harabasz index (Figure S4A), and could be classified as one of two types of enterotypes. Enterotype 1 was mainly composed of Firmicutes, and enterotype 2 was dominated by Bacteroidetes. The gut microflora of the FMT-Lpr mice was dominated by enterotype 1, while the FMT-Mpj mice and FMT-PBS mice had similar microbial enterotype compositions, with enterotype 2 occupying an advantage (Figure S4B), which further validated that the gut microbial structure of the FMT-Lpr mice was distinct from that of the FMT-Mpj mice and FMT-PBS mice.

Next, we performed metagenomic sequencing to provide deep insight into the taxonomic and structural differences among the three groups at T3, the timepoint at which the groups displayed significantly discrepant phenotypes and microbial structural distinctions as indicated by the 16S rRNA sequencing results. In fact, the results were further verified by PCoA on the basis of the metagenome data, revealing the separation of the sample distributions among the three groups (Figure S4C). Focusing on the microbiota taxonomic composition, we found that Bacteroides, Firmicutes, Actinobacteria, and Proteobacteria were still the four major phyla at the phylum level (Fig. 3B). The ratio of Firmicutes/Bacteroidetes increased gradually from FMT-Lpr mice to FMT-Mpj mice to FMT-PBS mice (Fig. 3C), which is consistent with previous results that a lower Firmicutes/Bacteroidetes ratio could be detected in SLE-related dysbiosis. At the genus level, *Bacteroides* and *Prevotella* were the dominant taxa with varied relative abundances among the three groups (Fig. 3D). According to the Kruskal‒Wallis test, the number of microbial taxa at the genus level distinguishing one group from the other two groups was 149 for FMT-Lpr mice, 35 for FMT-Mpj mice, and 118 for FMT-PBS mice. Incorporated with linear discriminant analysis (LDA), we screened out the representative genera for pristane-induced lupus mice with different fecal microbiota interventions (Fig. 3E). *Prevotella*, *Alloprevotella*, and *Bacteroides* were enriched in FMT-Lpr mice; *Candidatus_arthromitus* were enriched in FMT-Mpj mice, and *Adlercreutzia*, *Olsenella*, *Atopobium*, *Collinsella*, *Holdemania*, *Turicibacter*, *Streptococcus, and Bilophila,* and *Unclassified_f__Coriobacteriaceae* were enriched in FMT-PBS mice. Notably, among these representative taxa, the relative abundances of *Prevotella*, *Alloprevotella*, *Bilophila*, and *Streptococcus* exhibited gradually altering tendencies from FMT-Lpr mice to FMT-Mpj mice to FMT-PBS mice (Fig. 3F, Figure S5). By means of metagenome sequencing’s advantages in species-level bacterial classification, we found that several species belonging to the above characteristic genera were also meaningful as discriminative signatures for the three groups (Figure S6). In particular, several strains of *Prevotella* were predominantly increased in FMT-Lpr mice and may serve as microbial biomarkers of aggravated states of lupus.

### FMT led to a disordered metabolic profile in the pristane-induced lupus mice

**Fig. 4:**
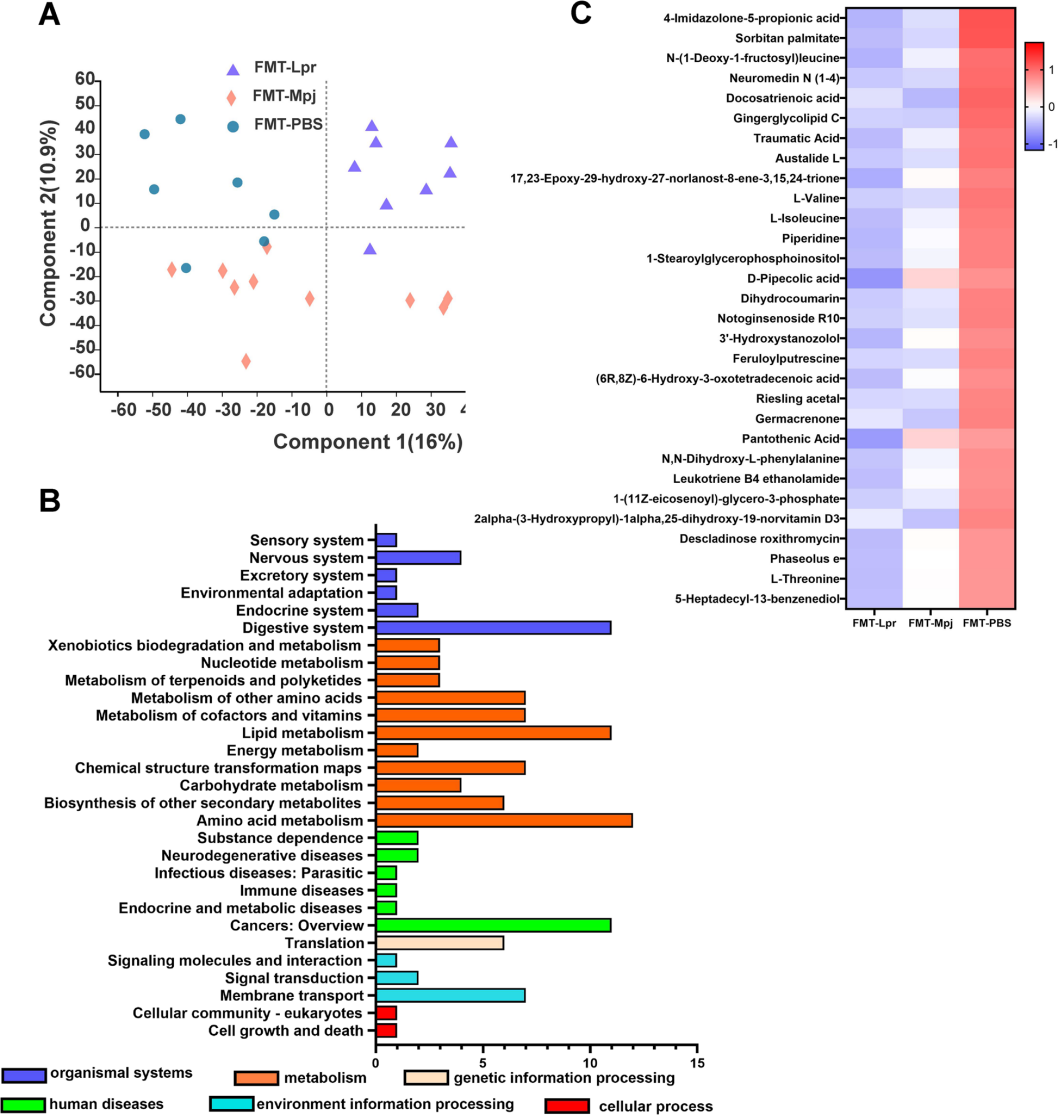
FMT altered the gut metabolite profiles.** A** Partial least squares discriminant analysis (PLS-DA) of fecal metabolomics data from three groups. Each dot represents one fecal sample. Different color represents different group (purple = FMT-Lpr, red = FMT-Mpj, blue = FMT-PBS). **B** The KEGG functional pathways enriched by differentiated metabolites. **C** The top 30 differentiated metabolites in the FMT-Lpr, FMT-Mpj, and FMT-PBS mice visualized on a heat map. FMT-Lpr, *n* = 11; FMT-Mpj, *n* = 10; FMT-PBS, *n* = 7

The metabolomes of fecal samples from the three groups were characterized and compared based on the LC‒MS platform. Distinct clusters related to pristane-induced lupus mice with different fecal microbiota transplantations could be visualized in the PLS-DA diagram, and the separations of the metabolic distributions among the three groups were significant (Fig. 4A). Metabolites that had distinctive levels among the three groups with *p* < 0.05 were mostly annotated in the metabolism pathway especially amino acid metabolism (Fig. 4B). The top 30 differentiated metabolites were presented through a heatmap, and we found that the levels of the majority of significant metabolites were reduced in the FMT-Lpr mice and elevated in the FMT-PBS mice while the FMT-Mpj mice showed transitional changing trends (Fig. 4C). Next, the significant metabolites with VIP scores > 1.0 in multivariate statistical analysis were compared in pairs and visualized in bubble diagrams respectively. Many metabolites could be used to distinguish pristane-induced lupus mice receiving different fecal microbiota interventions. For example, sorbitan stearate, alpha-D-galacturonic acid, and L-glutamate could differentiate FMT-Lpr mice from FMT-Mpj mice (Figure S7), sorbitan stearate, and L-isoleucine could differentiate FMT-Lpr mice from FMT-PBS mice (Figure S8), and linoleoyl ethanolamide, dihydrocoumarin, LPC, and L-valine could differentiate FMT-Mpj mice from FMT-PBS mice (Figure S9). The discriminative metabolites annotated in the KEGG pathway database are listed in Table S3, and some metabolites, such as L-valine, L-isoleucine, choline, and pantothenic acid, had differentiated relative abundances among the three groups, which represented different pathological states of the pristane-induced lupus mice. Therefore, these findings suggested that the metabolites of the gut microbiota may play important roles in the disease progression and pathophysiology of lupus.

### Characteristic functional pathways in the pristane-induced lupus mice with FMT

**Fig. 5:**
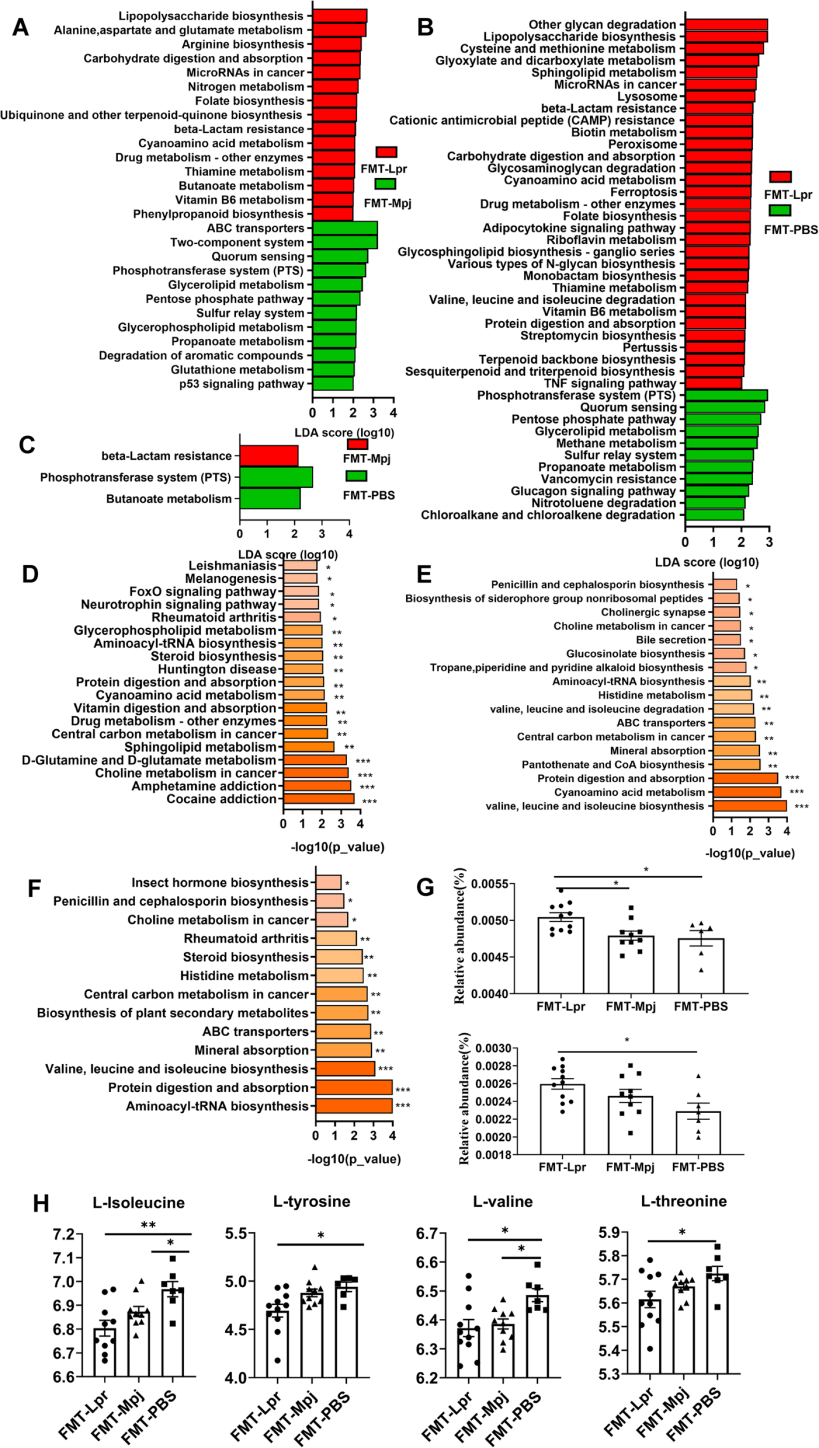
Differential enriched functional KEGG pathway in the pristane-induced lupus mice with different FMT. The characteristic KEGG functional pathways for each group based on the metagenome results were analyzed in the pairwise comparison, shown by LDA score > 2.0 between the FMT-Lpr and FMT-Mpj (**A**), the FMT-Lpr and FMT-PBS (**B**), and the FMT-Mpj and FMT-PBS (**C**). The KEGG functional pathway enriched by the differential metabolites analyzed in pairs between the FMT-Lpr and FMT-Mpj (**D**), the FMT-Lpr and FMT-PBS (**E**), the FMT-Mpj and FMT-PBS (**F**). **G** The relative abundances of the overlapped KEGG pathways including the cyanoamino acid metabolism (upper), valine, leucine, and isoleucine degradation (down) among the three groups. **H** The relative abundances of the L-isoleucine, L-valine, L-glutamate, L-tyrosine, and L-threonine among the three groups. *p < 0.05. **p < 0.001. FMT-Lpr, *n* = 11; FMT-Mpj, *n* = 10; FMT-PBS, *n* = 7

We found that the gut microbial and metabolite components were significantly different in pristane-induced lupus mice under FMT. It is necessary to investigate how these microbiota and metabolites are interrelated and affect the functional variations in different microbial ecosystems. The significantly differentiated KEGG pathways with LDA scores > 2.0 based on the metagenome results were identified and compared among the three groups. The number of functional KEGG pathways enriched by differential microbiota in the pairwise comparison was 27 for differentiating the FMT-Lpr mice and FMT-Mpj mice (Fig. 5A), 42 for differentiating the FMT-Lpr mice and FMT-PBS mice (Fig. 5B), and 3 for differentiating the FMT-Mpj mice and FMT-PBS mice (Fig. 5C). The KEGG pathways enriched by differentiated fecal metabolites based on the metabolomics results were also analyzed in pairs, presented in the form of bar graphs ordered by the enriched *p* value (Fig. 5D–F). The overlapping pathways, which involved the roles of characteristic microbial taxa and fecal metabolites, were considered highly important in the role of lupus progression. Functional pathways such as cyanoamino acid metabolism, ABC transporters, and glycerophospholipid metabolism made sense in discriminating FMT-Lpr mice and FMT-Mpj mice. Cyanoamino acid metabolism; valine, leucine, and isoleucine degradation; and protein digestion and absorption were associated with disparities in FMT-Lpr mice and FMT-PBS mice. There were no significant KEGG pathways enriched by both differentiated microbes and metabolites between FMT-Mpj and FMT-PBS.

Remarkably, among these representative KEGG pathways, we observed a gradual decreasing tendency of the relative abundances of cyanoamino acid metabolism (*p*_FMT-Lpr VS FMT-Mpj_ = 0.0294; *p*_FMT-Lpr VS FMT-PBS_ = 0.0326), and valine, leucine, and isoleucine degradation (*p*_FMT-Lpr VS FMT-PBS_ = 0.0214) from the FMT-Lpr mice to FMT-Mpj mice to FMT-PBS mice (Fig. 5G, Figure S10). Significant metabolites such as L-isoleucine, L-valine, L-glutamate, L-tyrosine, and L-threonine participated in cyanoamino acid metabolism as well as valine, leucine, and isoleucine degradation. The abundances of these characteristic metabolites showed corresponding variation trends with the abundances of the two above pathways (L-isoleucine:* p*_FMT-Lpr VS FMT-PBS_ = 0.0022; *p*_FMT-Mpj VS FMT-PBS_ = 0.0221, L-valine:* p*_FMT-Lpr VS FMT-PBS_ = 0.0122; *p*_FMT-Mpj VS FMT-PBS_ = 0.0337. L-tyrosine:* p*_FMT-Lpr VS FMT-PBS_ = 0.0229; *p*_FMT-Lpr VS FMT-Mpj_ = 0.0527. L-threonine:* p*_FMT-Lpr VS FMT-PBS_ = 0.0399) (Fig. 5H). Therefore, amino acid metabolism, such as cyanoamino acid metabolism and valine, leucine, and isoleucine degradation, may be of great importance in promoting the development of SLE.

### Strong associations between Prevotella and characteristic functional pathways

**Fig. 6:**
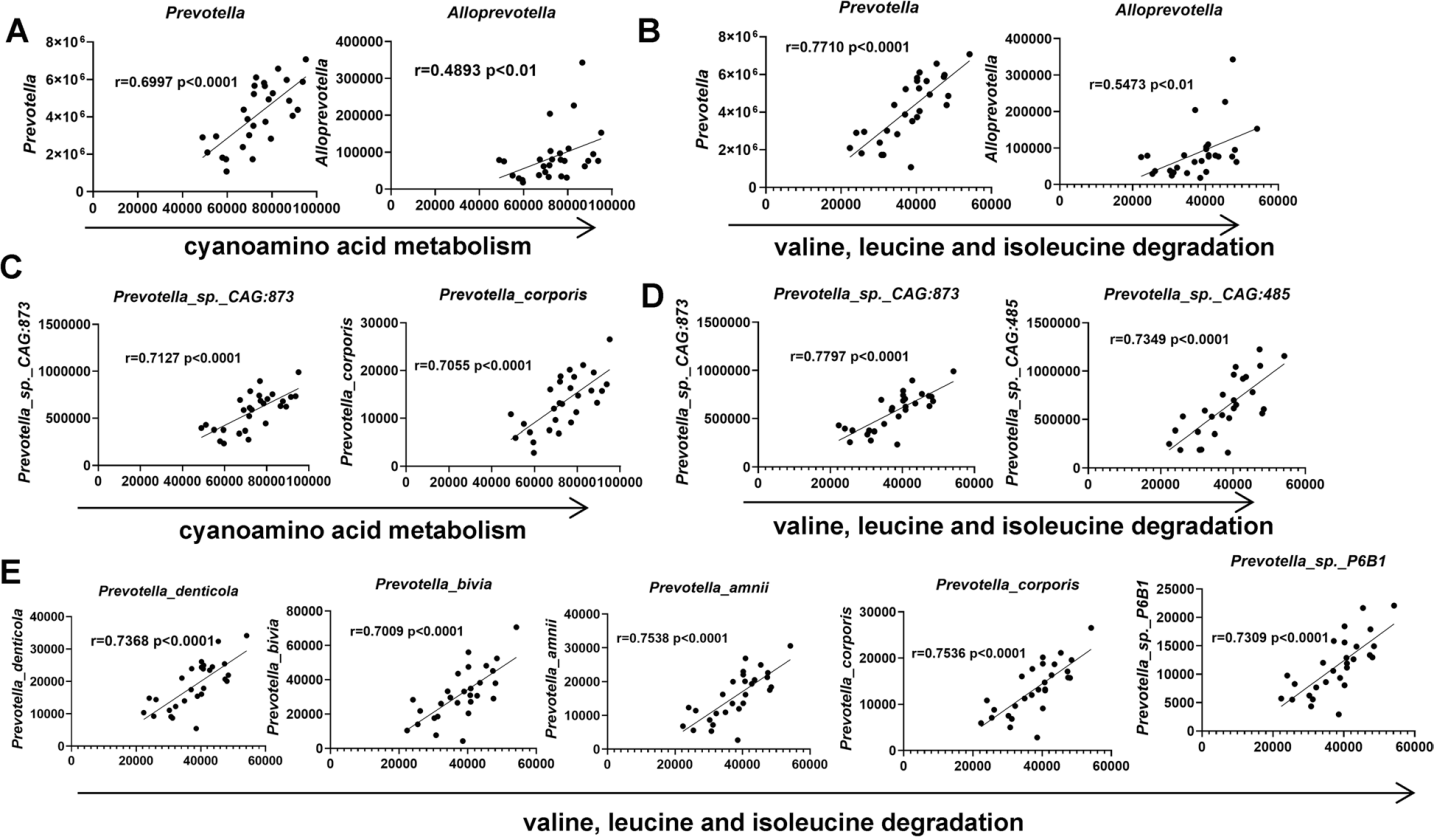
The associations between the abundances of characteristic taxa and disordered functional pathways.** A** The association of the abundances of *prevotella* and *alloprevotella* with the cyanoamino acid metabolism. **B** The association of the abundances of *prevotella* and *alloprevotella* with the valine, leucine, and isoleucine degradation. **C** The association of the abundances of *prevotella* species with the cyanoamino acid metabolism (correlation coefficient values > 0.7 are shown). **D, E** The association of the abundances of *prevotella* species with the valine, leucine, and isoleucine degradation (correlation coefficient values > 0.7 are shown). Spearman’s rank correlation coefficient, *r*-values, and *p*-values are shown. FMT-Lpr, *n* = 11; FMT-Mpj, *n* = 10; FMT-PBS, *n* = 7

Although we determined the representative taxa and the changed functional pathways accounting for the distinct phenotypes of the pristane-induced lupus mice due to the fecal microbiota transplantation from different strains of mice, we still needed to gain insight into how specific taxa acted in the characteristic metabolic pathways. Therefore, we investigated the correlations between the microbial taxa and the characteristic KEGG pathway. At the genus level, *Prevotella* and *Alloprevotella* were highly correlated with both cyanoamino acid metabolism (Fig. 6A) and valine, leucine, and isoleucine degradation (Fig. 6B). In particular, the correlation coefficients of *Prevotella* and the above two functional pathways were > 0.7 or almost reached 0.7, indicating strong correlations between *Prevotella* and cyanoamino acid metabolism and valine, leucine, and isoleucine degradation (Fig. 6A,B). Moreover, several strains of *Prevotella* were also positively related to the two characteristic pathways. The degree of the correlation was significant and strong, such as *Prevotella_sp._CAG:873* and *Prevotella_corporis* with cyanoamino acid metabolism (Fig. 6C) as well as *Prevotella_sp._CAG:873*, *Prevotella_sp._CAG:485*, *Prevotella_denticola*, *Prevotella_bivia*, *Prevotella_amni*, *Prevotella_corporis*, and *Prevotella_sp._P6B*1 with valine, leucine, and isoleucine degradation (Fig. 6D,E), which means that the levels of the discriminating functional pathways and the significant taxa, such as *Prevotella_sp._CAG:873* and *Prevotella_corporis*, were synchronously changed. Therefore, the abundance alterations in specific taxa may affect these microbiota and metabolite-associated functional pathways, thus contributing to the progression of lupus.

### The connections between the specific Prevotella taxa and the phenotypic changes in the pristane-induced lupus mice

**Fig. 7:**
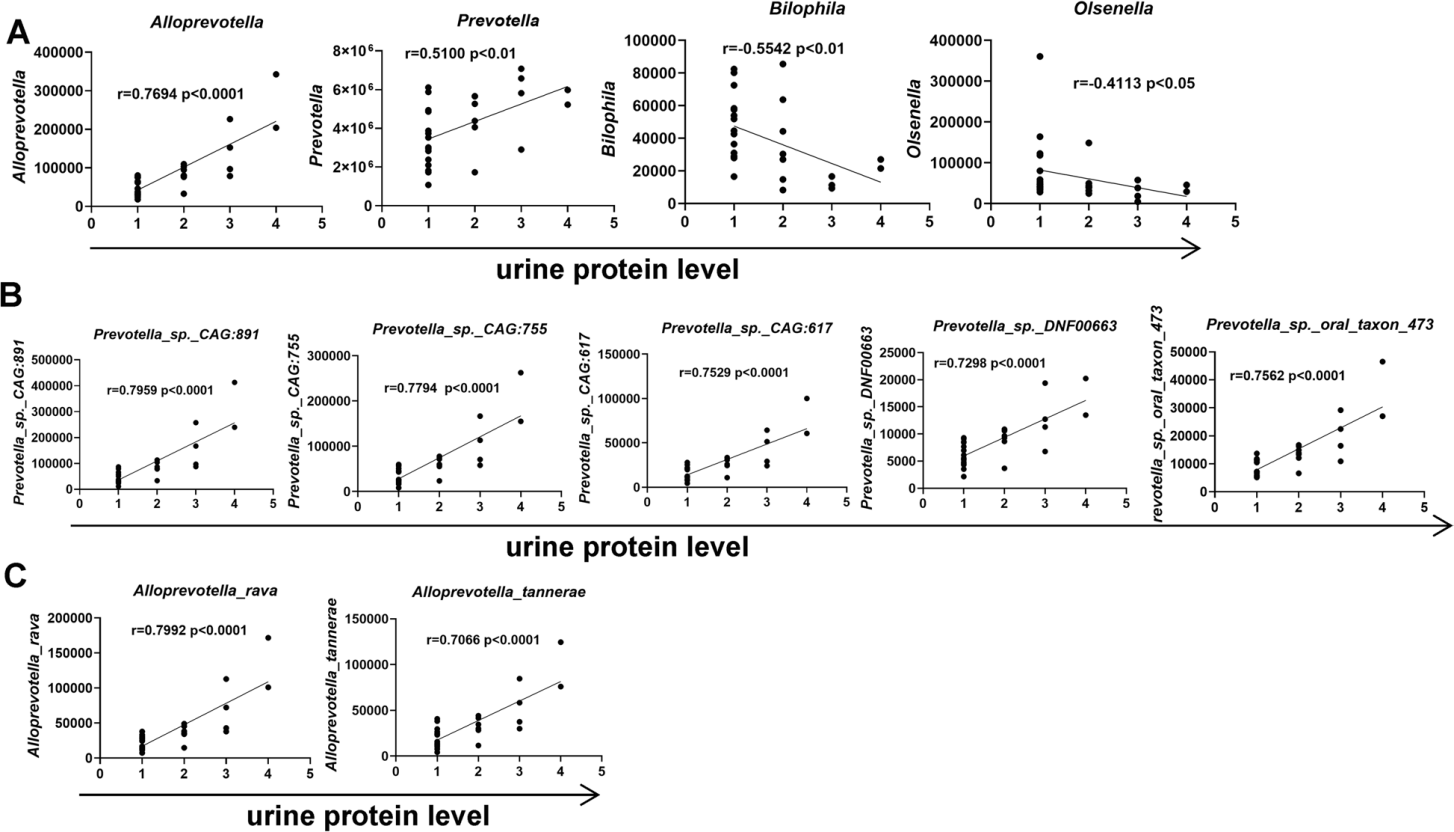
The associations between the urine protein levels and the abundances of characteristic taxa. **A** The association of the abundances of *prevotella*,* alloprevotella*,* bilophila*, and* oslenella* with the urine protein levels. **B** The association of the abundances of the specific *prevotella* taxa with the urine protein levels (correlation coefficient values > 0.7 are shown). **C** The association of the abundances of the specific *alloprevotella* taxa with the urine protein levels (correlation coefficient values > 0.7 are shown). Spearman’s rank correlation coefficient, *r*-values, and *p*-values are shown. FMT-Lpr, *n* = 11; FMT-Mpj, *n* = 10; FMT-PBS, *n* = 7

The pristane-induced lupus mice had different degrees of renal injuries, manifested as increased urine protein levels and more IgG and C3 deposition in the glomeruli of the mice receiving fecal microbiota from MRL/lpr mice compared with the FMT-Mpj mice and FMT-PBS mice. How the gut microbiota influences renal functions is still unknown. However, we found that the abundances of specific microbiota, such as *Prevotella*, *Alloprevotella*, *Bilophila*, and *Osenella*, were correlated with urine protein levels. In particular, the abundance of *Alloprevotella* showed a strong association with the levels of proteinuria (Fig. 7A). At the species level, the abundances of *Prevotella_sp._CAG:891*, *Prevotella_sp._CAG:755*, *Prevotella_sp._CAG:617*, *Prevotella_sp._oral_taxon_473*, *Prevotella_sp._DNF00663* (Fig. 7B), *Alloprevotella tannerae*, and *Alloprevotella_rava* (Fig. 7C) were also strongly positively related to the proteinuria levels. Therefore, these characteristic microbes may play roles in the progression of renal dysfunction.

Increased plasma cell percentages may be considered one of the predominant immunological features in the intestinal tissues and lymph nodes of the pristane-induced lupus mice. The *Prevotella* species exhibited connections with alterations in plasma cells to some degree. The abundances of *Prevotella_sp._CAG:891*, *Prevotella_sp._CAG:755*, *Prevotella_sp._CAG:5226*, *Prevotella_sp._oral_taxon_473*, and *Prevotella_sp._CAG:617* were associated with the plasma cell percentages in the large intestine (Figure S11A). The abundances of *Prevotella_bivia*, *Prevotella_sp._MA2016*, *Prevotella_corporis*, *Prevotella_stercorea*, *Prevotella_melaninogenica*, and *Prevotella_sp._P6B1* were associated with plasma cell levels in Peyer’s patches (Figure S11B). *Prevotella_bivia*, *Prevotella_stercorea*, and *Prevotella_buccae* were associated with the plasma cell alteration tendency in the mesenteric lymph nodes (Figure S11C). Taken together, the characteristic taxa, especially the strains of *Prevotella*, were possibly involved in the changed immunological states of lupus mice. Although it is still unclear whether the increased abundances of the specified taxa promoted lupus renal dysfunction or whether systemic phenotypic changes contributed to intestinal disorders, targeting specific taxa could be useful for disease therapies.

## Discussion

In this study, we investigated the effects of FMT on the disease development of pristane-induced lupus mice and found that feces from MRL/lpr mice could promote lupus progression. Based on the metagenomic and metabolomics results, we discovered that the gut microbial communities were gradually altered from FMT-PBS mice to FMT-Mpj mice to FMT-Lpr mice. Specific Prevotella taxa and metabolites, such as valine and L-isoleucine, were significantly altered in FMT-Lpr mice and could have great potential as therapeutic targets for SLE.

Compared with the pristane-induced lupus mice with PBS as the control group, although the alpha diversity remained unchanged after receiving FMT, there was a gradually clear separation of the microbial communities of the FMT-Lpr mice, FMT-Mpj mice, and FMT-PBS mice over time, which means that the structure and composition of the gut microbiome of pristane-induced lupus mice have been changed after the transfer of fecal microbiota, especially from the MRL/lpr mice. At the phylum level, Firmicutes, Bacteroides, Actinobacteria, and Proteobacteria are the four dominant phyla in the gut microbiome, which is consistent with previous reports of the gut microbial composition in SLE patients [24]. The decreased ratio of the Firmicutes/Bacteroidetes ratio could be considered as a potential indicator of gut dysbiosis in SLE patients [24, 25]. According to our results, the Firmicutes/Bacteroidetes ratio was further decreased in the FMT-Lpr mice, which suggests that the ratio could also be indicative of the lupus state. Taking advantage of the metagenome’s high resolution at the genus and species levels, we gained deep insight into the specific taxa for pristane-induced lupus mice under different conditions and found that the abundances of *Prevotella* species were significantly elevated in FMT-Lpr mice. The genus *Prevotella* is an SLE-enriched microorganism validated in Chinese populations [25]. However, it is still unclear how *Prevotella* species function to promote SLE pathogenesis.

*Prevotella* species, which are gram-negative bacteria, extensively exist in the human body and are deemed commensal bacteria in most cases. However, emerging evidence has shown that the increased abundances of *Prevotella* species are linked to several chronic inflammatory diseases and autoimmune diseases [26]. Pathobionts are capable of augmenting inflammatory mediators released from epithelial and immune cells, thus participating in localized and systemic inflammation [26]. In this study, pristane-induced lupus mice exhibited aggravated disease progression with the transplantation of fecal microbiota from MRL/lpr mice. *Prevotella* species exhibit more pathobiontic properties than commensal characteristics during lupus progression. It is universally deemed that *Prevotella* can stimulate the production of inflammatory cytokines and predominantly mediate the Th17-related response [27]. However, our data revealed that the mRNA levels of inflammatory cytokines such as IFN-γ, IL-6, and IL-18 were increased in the intestinal tissues of FMT-Lpr mice, which was mainly characterized as plasma cell-related localized and systemic immunity. The increased abundances of *Prevotella* and several strains of *Prevotella* were also positively associated with proteinuria levels, implicating the potential role of *Prevotella* species in the gut-kidney axis and even impairment of multiple organ systems in SLE. Recent research found that *Prevotella copri*-specific antibody production may contribute to the initiation or progression of rheumatoid arthritis [28], revealing the B cell-related immune responses could be also involved in prevotella-associated disease risk and progression. Some intestinal bacteria with orthologue Ro60 sequences homologous to hRo60, such as *s__Prevotella_sp._CAG:1092*, equipped with the common Ro60 B-cell epitope, allow for B-cell cross-reactivity between human and bacterial Ro60, thus triggering and maintaining chronic autoimmunity in individuals with genetic predisposition [29]. Therefore, further diving into the intrinsic correlations between *Prevotella* species and local and systemic immune responses in lupus is warranted.

Furthermore, our results revealed that amino acid metabolism, especially the pathways of cyanoamino acid metabolism as well as valine, leucine, and isoleucine degradation, was significantly elevated in FMT-Lpr mice. Notably, these pathways are also altered in the fecal metabolome of SLE patients compared with healthy controls [30, 31]. The abundances of *Prevotella* species were positively associated with cyanoamino acid metabolism as well as valine, leucine, and isoleucine degradation, which originate from substrates such as valine, L-isoleucine, and L-tyrosine and promote the degradation of these compounds. Indeed, the abundances of valine, L-isoleucine, and L-tyrosine were significantly depleted in FMT-Lpr mice. Therefore, specific *Prevotella* taxa possibly influence the gut metabolome and subsequently amplify alterations during SLE disease progression.

Valine, L-isoleucine, and L-tyrosine are important essential amino acids that play crucial roles in the physiological activities of both hosts and microorganisms. The imbalance of these amino acids could induce many health issues, such as diabetes and cancers [32]. Valine and isoleucine can enhance the gut health by promoting intestinal development, nutrient transporters, and disease resistance [33–35]. On the one hand, the deficiency of valine or leucine cannot provide enough fuel, thus impairing immune functions due to the shortage of immune cells [36, 37]. On the other hand, branched-chain amino acid (BCAA) treatments, including valine and leucine, stimulate the mRNA expression of β-defensins, occludin, and claudin in the intestine [37], which are important for intestinal barrier functions. Our results revealed that the expression of the tight junction protein ZO-1 in the intestine was significantly decreased in FMT-Lpr mice, indicating destruction of the intestinal barriers, which is possibly ascribed to the reduced levels of valine and leucine. Decreased intestinal permeability results in bacterial translocation or the leakage of bacterial products, which is important for systemic autoimmunity and lupus progression, including autoantibody production and the development of lupus nephritis. Thus, this pathway may explain the aggravated phenotypes in FMT-Lpr mice. In fact, several studies have reported that multiple amino acids, including valine, L-isoleucine, and L-tyrosine, were depleted in SLE patients compared with healthy volunteers based on serum metabolomics results [38–40]. However, a recent report focused on the fecal metabolome of SLE patients and drew the opposite conclusion that valine, L-isoleucine, and L-tyrosine were significantly enriched in SLE feces [31]. This difference may be due to the distinct gut bacterial spectra and metabolite compositions in mice and humans. In addition, our data were obtained by LC‒MS methods, while the other reports relied on the GC‒MS platform. The different employment of analysis methods may also influence the disparities in the results. Overall, the causal relationships between the characteristic metabolites, including the tendencies and mechanisms of the alterations, and SLE pathogenesis should be further delineated in cohort studies.

## Conclusion

To summarize, our research has verified that fecal microbiota transplantation from MRL/lpr mice could change the intestinal immunological profiles as well as the structure and composition of microbial communities, thus promoting the disease development of pristane-induced lupus mice. Targeting the specific *Prevotella* species or supplementing amino acids such as valine and L-isoleucine may be effective for improving the symptoms and suppressing the disease progression in lupus, which lays a foundation for clinical translations such as microbiota or metabolite-directed therapies for SLE patients in the future. However, this research has several limitations. First, due to the complexities of sampling and tissue digestion as well as cell type diversity in the intestines, whether FMT could affect the number and the percentage of CD45 + cells could not be clearly determined; thus this should be evaluated in the future. Second, it is still unclear how the prevotella species promote lupus progression, especially the functional mechanisms affecting the immunological microenvironment. Third, it remains to be further deciphered how the prevotella species and characteristic metabolites interact and contribute to the development of SLE. Last but not least, potential biomarkers, such as the prevotella species, should be validated in clinical practice. In summary, whether the abundant alterations of the prevotella taxa are indicative of the severity of the lupus warrants further investigation.

## Supplementary Information

**Additional file 1: Supplementary Table 1.** Primer Sequences for real-time PCR. **Supplementary Table2.** The alpha diversity of the FMT-Lpr, FMT-Mpj and FMT-PBS mice at different time points. **Supplementary Table 3.** The significantly differentiated metabolites with annotations in the KEGG databases in the pairwise comparisons among the FMT-Lpr, FMT-Mpj and FMT-PBS mice. **Supplementary Figure 1.** (A) Representative flow diagram of gating strategies for T and B lymphocytes in the spleen and lymph nodes. (B) Representative flow diagram of gating strategies for T and B lymphocytes in the kidney. (C) Representative flow diagram of gating strategies for T cells, B cells, innate lymphoid cells (ILCs) as well as the CD11b+ cell subsets in the large and small intestine. **Supplementary Figure 2.** (A) mRNA expression levels of the inflammatory cytokines in the kidney (Il6, Tnf, Il17a, Ifng). (B) Flow cytometric analysis of the CD4+, CD8+ cells gated on the CD3+ T cells, B220+ cells and plasma cells gated on the live cells in the kidney. (C) Serum anti-dsDNA IgG quantified by ELISA. (D) Flow cytometric analysis of the Th1, Th2, Th17 percentages gated on the CD4+ T cell in the spleens and lymph nodes (SP: spleen; DLN: draining lymph node; MLN: mesentery lymph nodes; PP: peyer’s patch). *p < 0.05.**p<0.001. FMT-Lpr, *n*=10~11; FMT-Mpj, *n*=9~10; FMT-PBS, *n*=6~7. **Supplementary Figure 3.** mRNA expression levels of IL-6, IL-12a, IL-17a in the small intestine (A) and IL-12a, IL-17a in the large intestine (B). Flow cytometric analysis of the ILC1, ILC2, ILC3 subsets percentages gated on the CD45+CD3-CD19-CD127+ cells in the small intestine (C) and the large intestine (D). Flow cytometric analysis of the CD4+ and CD8+ cell percentages gated on the CD3+ cells in the small intestine (E) and the large intestine (F). Flow cytometric analysis of the CD44+CD62L- cell percentages gated on the CD4+ T cell (G) and CD8+T cell (H). (Left: the small intestine; right: the large intestine). (I) Flow cytometric analysis of the CD11b+ cell percentages gated on the CD45+CD3-CD19- cells in the large intestine. (J) Flow cytometric analysis of the Ly6C-Ly6G+ and Ly6C+Ly6G- cell percentage gated on the CD11b+ cells in the large intestine. *p < 0.05.**p< 0.001. FMT-Lpr, *n*=8~11; FMT-Mpj, *n*=5~10; FMT-PBS, *n*=5~7. **Supplementary Figure 4.** (A) The Calinski-Harabasz index plot indicated two was the optimal number for clustering the enterotypes. (B) The compositions of the two enterotypes in each group (blue: enterotype1, green: enterotype2). (C) Principal coordinate analysis (PCoA) of the gut microbial community based on the metagenome data. **Supplementary Figure 5.** The relative abundances of *Candidatus_Arthromitus, Adlercreutzia, Olsenella, Atopobium, Collinsella, Holdemania, unclassified_f__Coriobacteriaceae, Turicibacter* among three groups. *p < 0.05.**p<0.001. FMT-Lpr, *n*=11; FMT-Mpj, *n*=10; FMT-PBS, *n*=7. **Supplementary Figure 6.** Differential bacterial taxa for each group shown by LDA score> 2.0 at the species level. **Supplementary Figure 7.** The VIP bubble plot showed the differential metabolites ordered by the VIP scores and the corresponding expression heatmap between the FMT-Lpr and FMT-Mpj. The deeper the color is, the higher the level of the metabolite is. **Supplementary Figure 8.** The VIP bubble plot showed the differential metabolites ordered by the VIP scores and the corresponding expression heatmap between the FMT-Lpr and FMT-PBS. The deeper the color is, the higher the level of the metabolite is. **Supplementary Figure 9.** The VIP bubble plot showed the differential metabolites ordered by the VIP scores and the corresponding expression heatmap between the FMT-Mpj and FMT-PBS. The deeper the color is, the higher the level of the metabolite is. **Supplementary Figure 10.** The relative abundances of the ABC transporters, Protein digestion and absorption, Glycerophospholipid metabolism among the FMT-Lpr, FMT-Mpj and FMT-PBS. *p < 0.05.**p<0.001. FMT-Lpr, *n*=11; FMT-Mpj, *n*=10; FMT-PBS, *n*=7. **Supplementary Figure 11.** (A) The association of the abundances of the *prevotella* taxa with the percentage of plasma cells in the large intestine. (B)The association of the abundances of the *prevotella* taxa with the percentage of plasma cells in the peyer’s patches. (C)The association of the abundances of the *prevotella* taxa with the percentage of plasma cells in the mesentery lymph nodes. only *r* values>0.4 and *p* values<0.05 were shown.

## Data Availability

Raw data were deposited in the NCBI SRA (BioProject: PRJNA784741 for metagenomic sequencing data; Bioproject: PRJNA784655 for 16 s rRNA sequence data). Other analyzed data are included in this article and its additional files.
