# Supplementary material for: Fecal microbiota from MRL/lpr mice exacerbates pristane-induced lupus
Source: Arthritis Res Ther. 2023 Mar 16;25:42. doi: 10.1186/s13075-023-03022-w (PMC10018936; doi:10.1186/s13075-023-03022-w)
Supplement: Supplementary file 1 — Additional file 1: Supplementary Table 1. Primer Sequences for real-time PCR. Supplementary Table2. The alpha diversity of the FMT-Lpr, FMT-Mpj and FMT-PBS mice at different time points. Supplementary Table 3. The significantly differentiated metabolites with annotations in the KEGG databases in the pairwise comparisons among the FMT-Lpr, FMT-Mpj and FMT-PBS mice. Supplementary Figure 1. (A) Representative flow diagram of gating strategies for T and B lymphocytes in the spleen and lymph nodes. (B) Representative flow diagram of gating strategies for T and B lymphocytes in the kidney. (C) Representative flow diagram of gating strategies for T cells, B cells, innate lymphoid cells (ILCs) as well as the CD11b+ cell subsets in the large and small intestine. Supplementary Figure 2. (A) mRNA expression levels of the inflammatory cytokines in the kidney (Il6, Tnf, Il17a, Ifng). (B) Flow cytometric analysis of the CD4+, CD8+ cells gated on the CD3+ T cells, B220+ cells and plasma cells gated on the live cells in the kidney. (C) Serum anti-dsDNA IgG quantified by ELISA. (D) Flow cytometric analysis of the Th1, Th2, Th17 percentages gated on the CD4+ T cell in the spleens and lymph nodes (SP: spleen; DLN: draining lymph node; MLN: mesentery lymph nodes; PP: peyer’s patch). *p < 0.05.**p<0.001. FMT-Lpr, n=10~11; FMT-Mpj, n=9~10; FMT-PBS, n=6~7. Supplementary Figure 3. mRNA expression levels of IL-6, IL-12a, IL-17a in the small intestine (A) and IL-12a, IL-17a in the large intestine (B). Flow cytometric analysis of the ILC1, ILC2, ILC3 subsets percentages gated on the CD45+CD3-CD19-CD127+ cells in the small intestine (C) and the large intestine (D). Flow cytometric analysis of the CD4+ and CD8+ cell percentages gated on the CD3+ cells in the small intestine (E) and the large intestine (F). Flow cytometric analysis of the CD44+CD62L- cell percentages gated on the CD4+ T cell (G) and CD8+T cell (H). (Left: the small intestine; right: the large intestine). (I) Flow cytometric anal [file 13075_2023_3022_MOESM1_ESM.docx]

**Supplementary Table 1 Primer Sequences for real-time PCR**

| Primer | Forward (5’-3’) | Reverse (5’-3’) |
| --- | --- | --- |
| Il6 | CTCCCAACAGACCTGTCTATAC | CCATTGCACAACTCTTTTCTCA |
| Il18 | CAAAGTGCCAGTGAACCCCAGAC | ACAGAGAGGGTCACAGCCAGTC |
| Ifng | TGCTTTGCGTTGGACATTCAAG | TGCTTTGCGTTGGACATTCAAG |
| Il17a | TTCACTTTCAGGGTCGAGA | GGGGTTTCTTAGGGGTCA |
| Il12a | CAATCACGCTACCTCCTCTTTT | CAGCAGTGCAGGAATAATGTTTC |
| Tnf | ATGTCTCAGCCTCTTCTCATTC | GCTTGTCACTCGAATTTTGAGA |
| Actb | TATGCTCTCCCTCACGCCATCC | GTCACGCACGATTTCCCTCTCAG |
| ZO1 | TGTCAGGCATTGGCTAGAGGTTC | GGCCAAGGCCATAATAAACTTTGAG |

**Supplementary Table2: The alpha diversity of the FMT-Lpr, FMT-Mpj and FMT-PBS mice at different time points.**

| Estimators |  | FMT-Lpr  Mean±Sd | FMT-Lpr  Mean±Sd | FMT-PBS  Mean±Sd | P value  Lpr VS Mpj | P value  Lpr VS PBS | P value  Mpj VS PBS |
| --- | --- | --- | --- | --- | --- | --- | --- |
| shannon | T1 | 2.0956±0.34991 | 1.992±0.30401 | 2.1492±0.24198 | 0.3787 | 1 | 0.4068 |
|  | T2 | 4.2713±0.23541 | 4.4215±0.2808 | 4.43±0.15854 | 0.3072 | 0.239 | 0.5914 |
|  | T3 | 4.3323±0.28202 | 4.4011±0.24 | 4.4061±0.3021 | 0.6472 | 0.7172 | 0.8836 |
| simpson | T1 | 0.22336±0.10315 | 0.24139±0.086449 | 0.20985±0.05649 | 0.6985 | 0.7172 | 0.5914 |
|  | T2 | 0.039434±0.011804 | 0.035567±0.019265 | 0.030695±0.0072916 | 0.3787 | 0.07009 | 0.6605 |
|  | T3 | 0.040438±0.011916 | 0.035598±0.012036 | 0.033944±0.015862 | 0.5035 | 0.2048 | 0.5914 |
| chao | T1 | 161.65±71.182 | 83.373±37.57 | 90.36±32.393 | 0.002765 | 0.006588 | 0.4347 |
|  | T2 | 644.7±51.922 | 681.75±22.509 | 659.57±36.808 | 0.05281 | 0.7172 | 0.1571 |
|  | T3 | 638.63±50.02 | 661.87±41.829 | 646.32±34.204 | 0.5974 | 0.7172 | 0.4068 |

T1: the first week after the intraperitoneal injection of the pristane.

T2: the 5th month, the time point when the urine protein levels started to elevate.

T3: the 9th month, at the end of the experiment.

**Supplementary Table 3. The significantly differentiated metabolites with annotations in the KEGG databases in the pairwise comparisons among the FMT-Lpr, FMT-Mpj and FMT-PBS mice.**

| FMT-Lpr VS FMT-Mpj | FMT-Lpr VS FMT-PBS | FMT-Mpj VS FMT-PBS |
| --- | --- | --- |
| L-Glutamate  Choline  Hypoxanthine  Pantothenic Acid  Uridine  Isonicotinic acid  LysoPC(18:0)  7-dehydrocholesterol  3-ketosphinganine  4-Hydroxy-L-glutamic acid  L-Tyrosine  Prenyl glucoside  N-Stearoylsphingosine  Hydrocortisone cypionate  Glucosamine  Phaseolic acid  12S-HHT  LysoPC(P-18:1(9Z))  Ricinoleic acid  Enterodiol  Gamma-Glutamylglutamic acid  Acetyl-DL-Leucine  LysoPC(20:0)  Asparagoside A  3-Hydroxypicolinic acid  (S,E)-Zearalenone  Porphobilinogen  Lacto-N-biose I  N-(2-Phenylethyl)-acetamide | L-Isoleucine  Dihydrocoumarin  Choline  Piperidine  Pantothenic Acid  L-Valine  4-Imidazolone-5-propionic acid  Traumatic Acid  Prenyl glucoside  2,3-Dihydroxybenzoic acid  2-Isopropylmalic acid  6-Methoxymellein  N,N-Dihydroxy-L-phenylalanine  N-Alpha-acetyllysine  2-Hexaprenyl-6-methoxyphenol  Acetyl-DL-Leucine  12S-HHT  Feruloylputrescine  Acetyl-L-tyrosine  1-Methylhistidine  Asparagoside A  Deoxycholic acid 3-glucuronide  5-Acetamidovalerate  Lacto-N-biose I  7alpha-Hydroxy-3-oxo-4-cholestenoate  3-Carboxy-4-methoxy-N-methyl-2-pyridone | Linoleoyl Ethanolamide  Dihydrocoumarin  L-Valine  Drostanolone  4-Imidazolone-5-propionic acid  7-dehydrocholesterol  LysoPC(20:4(5Z,8Z,11Z,14Z))  Traumatic Acid  LysoPC(20:3(5Z,8Z,11Z))  L-Threonine  L-Histidine  4-Hydroxy-L-glutamic acid  Caryophyllene epoxide  Feruloylputrescine  Ricinoleic acid  Indole-3-carboxaldehyde |

**
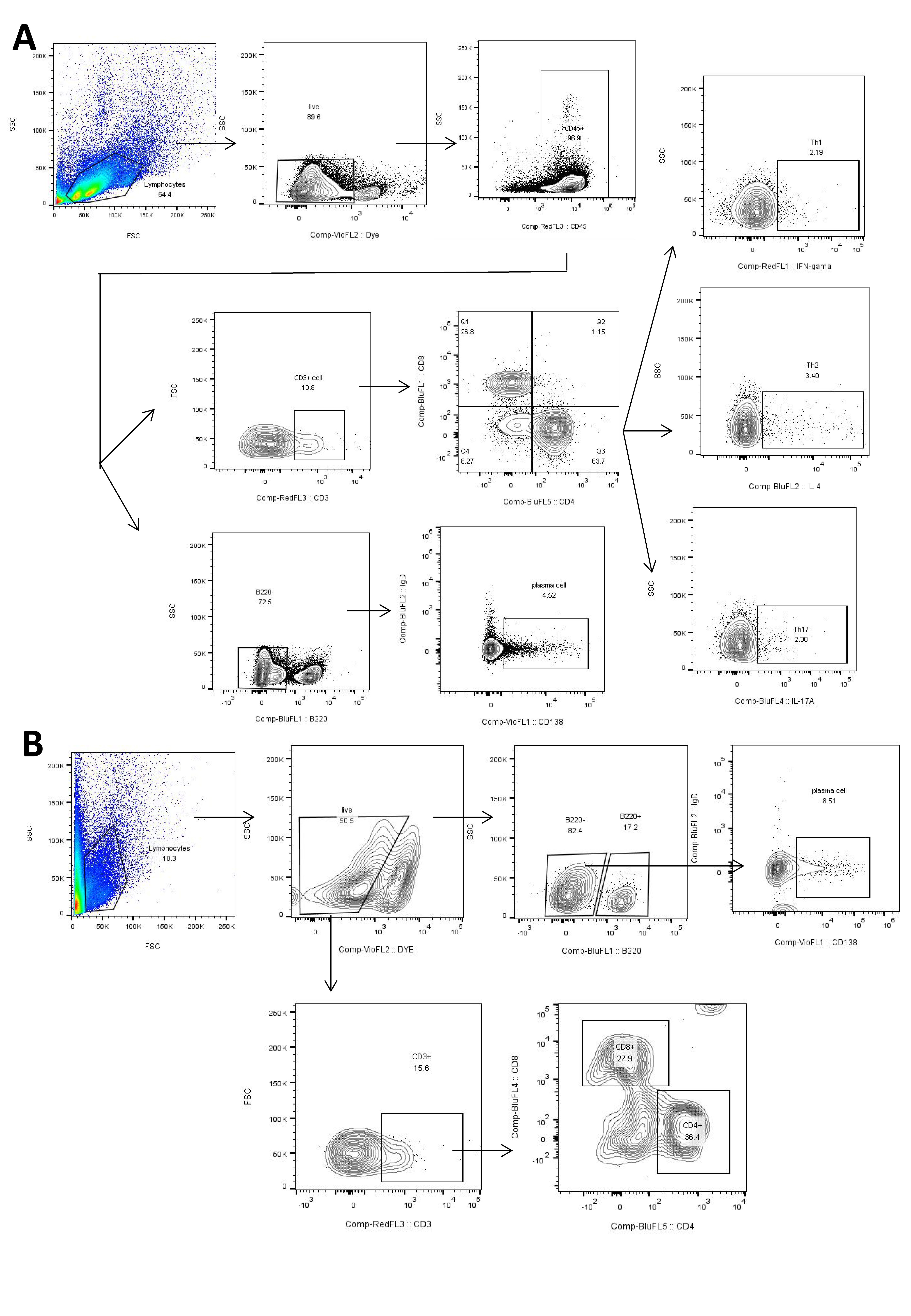

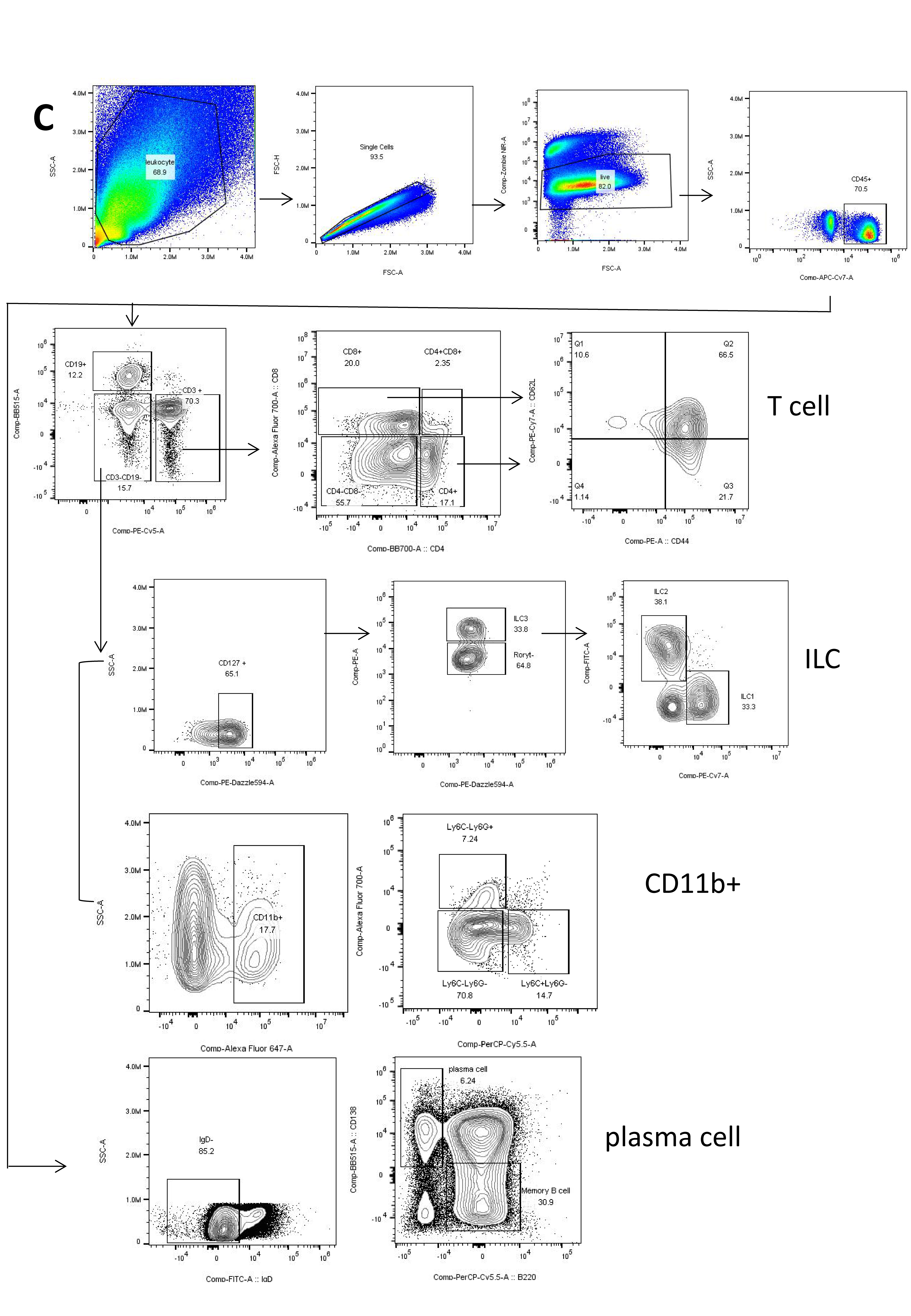
Supplementary Figure 1.** (A) Representative flow diagram of gating strategies for T and B lymphocytes in the spleen and lymph nodes. (B) Representative flow diagram of gating strategies for T and B lymphocytes in the kidney. (C) Representative flow diagram of gating strategies for T cells, B cells, innate lymphoid cells (ILCs) as well as the CD11b+ cell subsets in the large and small intestine.


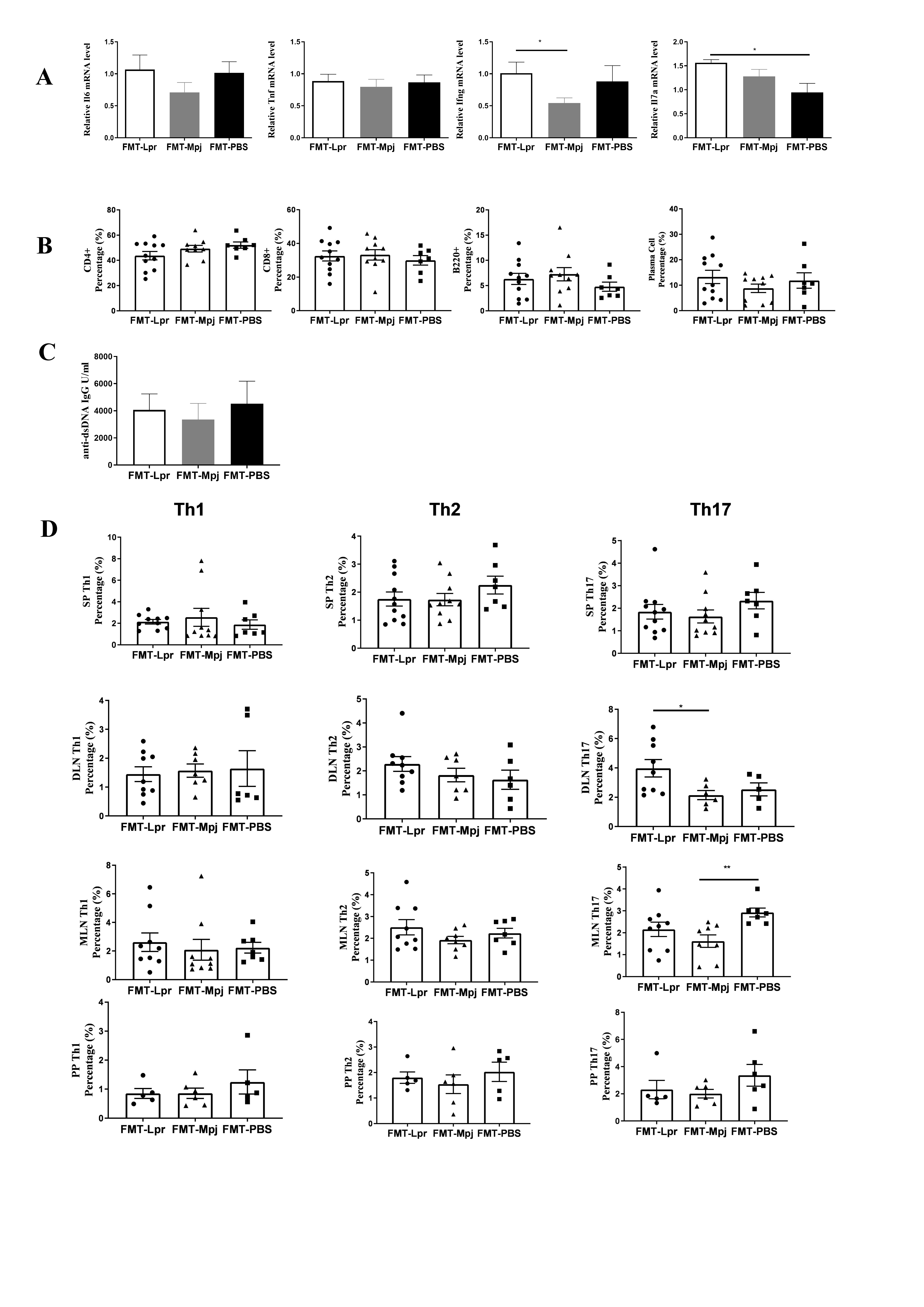


**Supplementary Figure 2.** (A) mRNA expression levels of the inflammatory cytokines in the kidney (Il6, Tnf, Il17a, Ifng). (B) Flow cytometric analysis of the CD4+, CD8+ cells gated on the CD3+ T cells, B220+ cells and plasma cells gated on the live cells in the kidney. (C) Serum anti-dsDNA IgG quantified by ELISA. (D) Flow cytometric analysis of the Th1, Th2, Th17 percentages gated on the CD4+ T cell in the spleens and lymph nodes (SP: spleen; DLN: draining lymph node; MLN: mesentery lymph nodes; PP: peyer’s patch). *p < 0.05.**p<0.001. FMT-Lpr, n=10~11; FMT-Mpj, n=9~10; FMT-PBS, n=6~7.


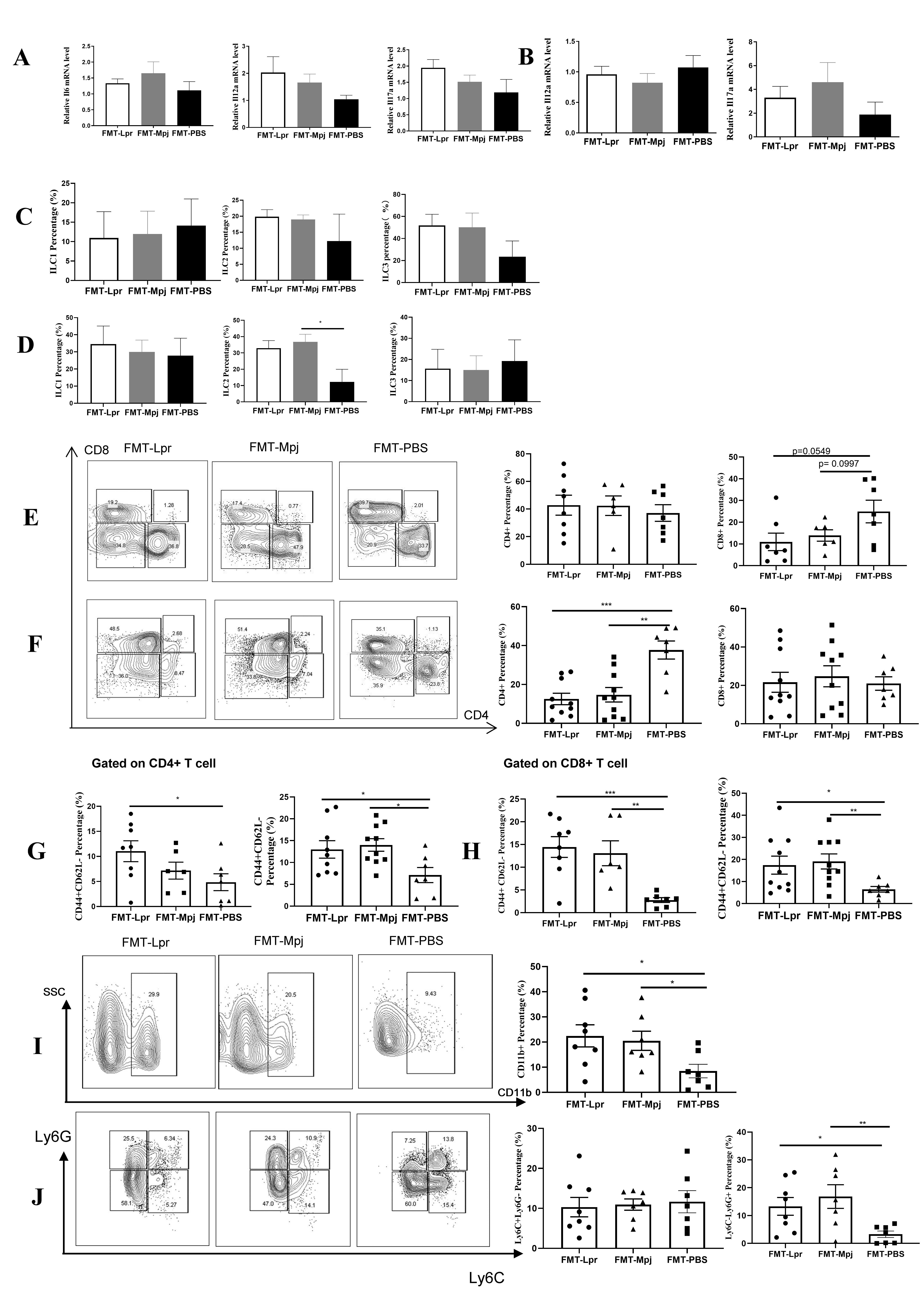


**Supplementary Figure 3.** mRNA expression levels of IL-6, IL-12a, IL-17a in the small intestine (A) and IL-12a, IL-17a in the large intestine (B). Flow cytometric analysis of the ILC1, ILC2, ILC3 subsets percentages gated on the CD45+CD3-CD19-CD127+ cells in the small intestine (C) and the large intestine (D). Flow cytometric analysis of the CD4+ and CD8+ cell percentages gated on the CD3+ cells in the small intestine (E) and the large intestine (F). Flow cytometric analysis of the CD44+CD62L- cell percentages gated on the CD4+ T cell (G) and CD8+T cell (H). (Left: the small intestine; right: the large intestine). (I) Flow cytometric analysis of the CD11b+ cell percentages gated on the CD45+CD3-CD19- cells in the large intestine. (J) Flow cytometric analysis of the Ly6C-Ly6G+ and Ly6C+Ly6G- cell percentage gated on the CD11b+ cells in the large intestine. *p < 0.05.**p< 0.001. FMT-Lpr, n=8~11; FMT-Mpj, n=5~10; FMT-PBS, n=5~7.


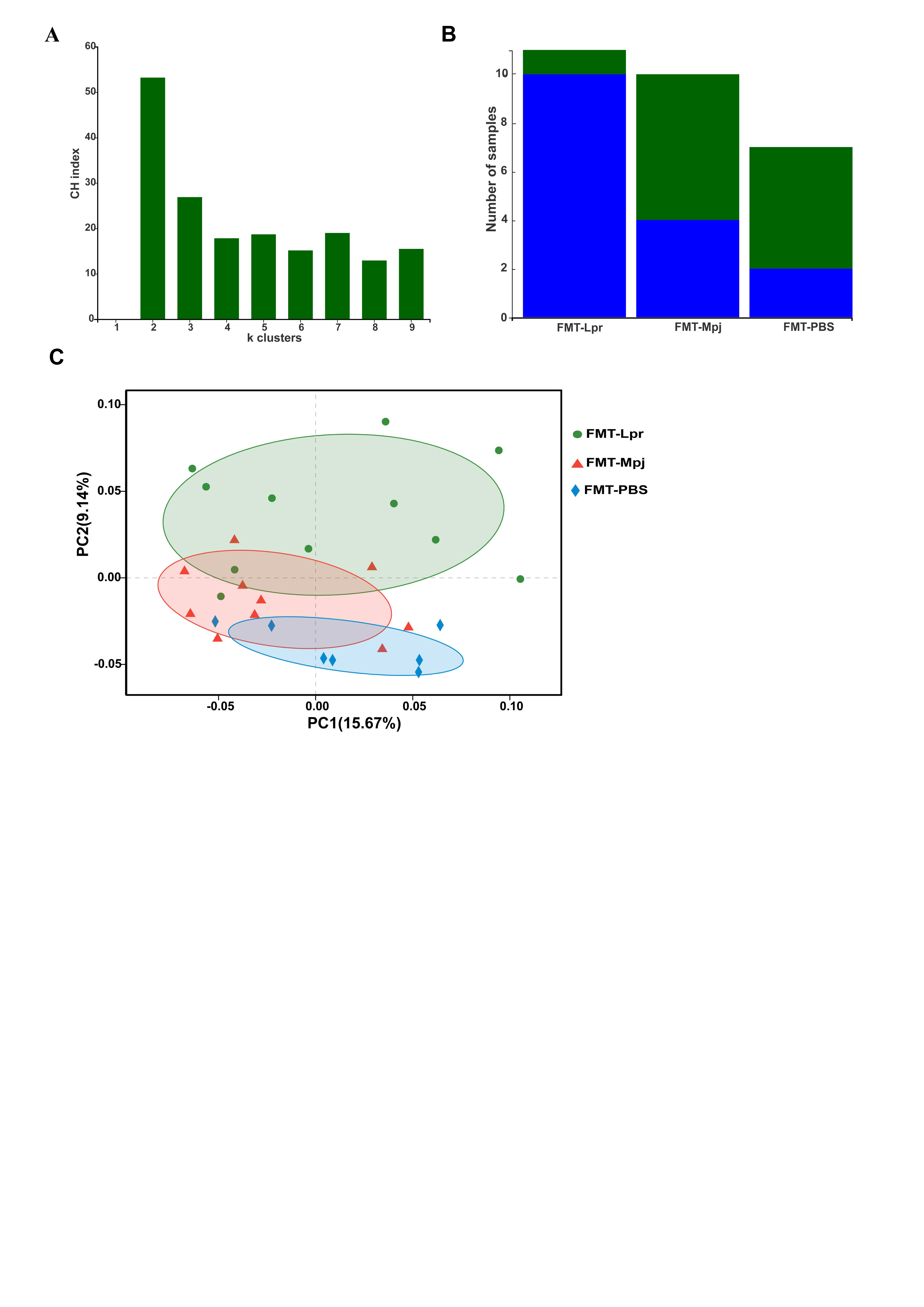


**Supplementary Figure 4.** (A) The Calinski-Harabasz index plot indicated two was the optimal number for clustering the enterotypes. (B) The compositions of the two enterotypes in each group (blue: enterotype1, green: enterotype2). (C) Principal coordinate analysis (PCoA) of the gut microbial community based on the metagenome data.

**Supplementary Figure 5.** The relative abundances of *Candidatus_Arthromitus, Adlercreutzia, Olsenella, Atopobium, Collinsella, Holdemania, unclassified_f__Coriobacteriaceae, Turicibacter* among three groups. *p < 0.05.**p<0.001. FMT-Lpr, n=11; FMT-Mpj, n=10; FMT-PBS, n=7.


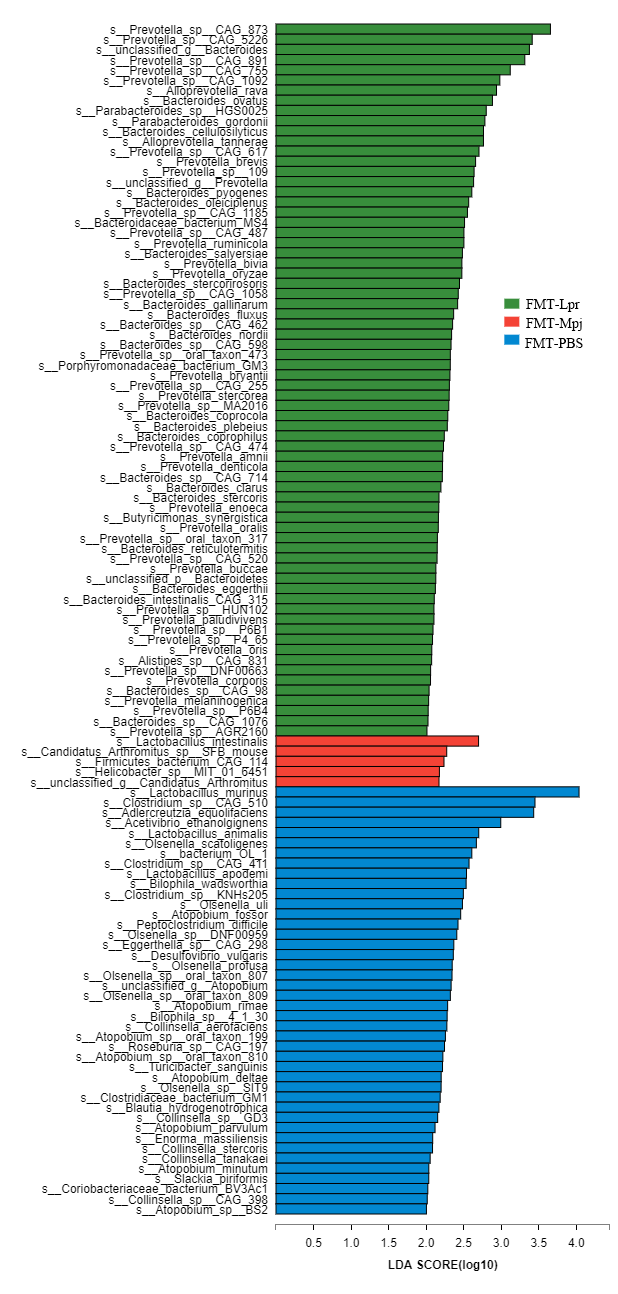


**Supplementary Figure 6.** Differential bacterial taxa for each group shown by LDA score> 2.0 at the species level.


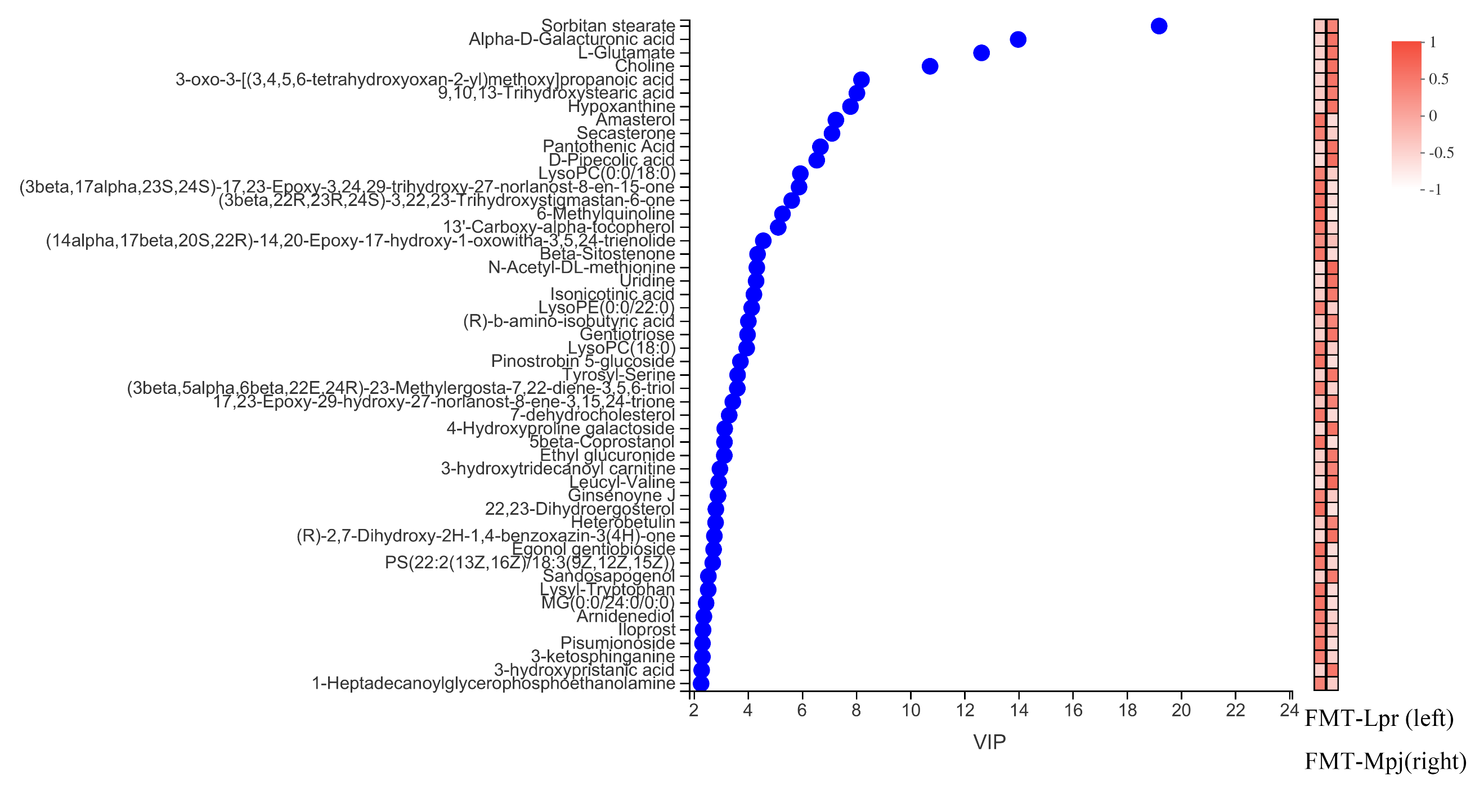


**Supplementary Figure 7.** The VIP bubble plot showed the differential metabolites ordered by the VIP scores and the corresponding expression heatmap between the FMT-Lpr and FMT-Mpj. The deeper the color is, the higher the level of the metabolite is.


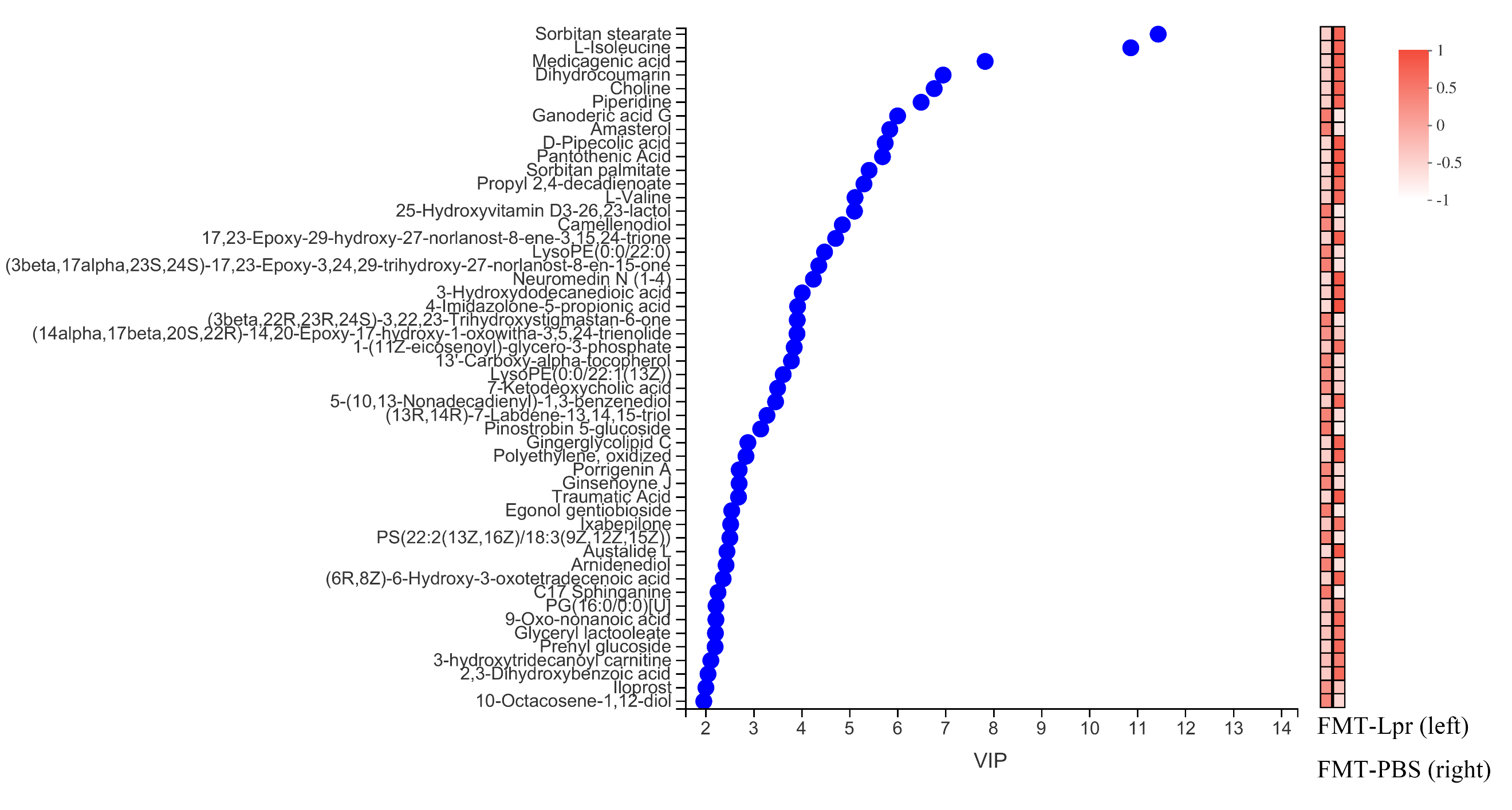


**Supplementary Figure 8.** The VIP bubble plot showed the differential metabolites ordered by the VIP scores and the corresponding expression heatmap between the FMT-Lpr and FMT-PBS. The deeper the color is, the higher the level of the metabolite is.


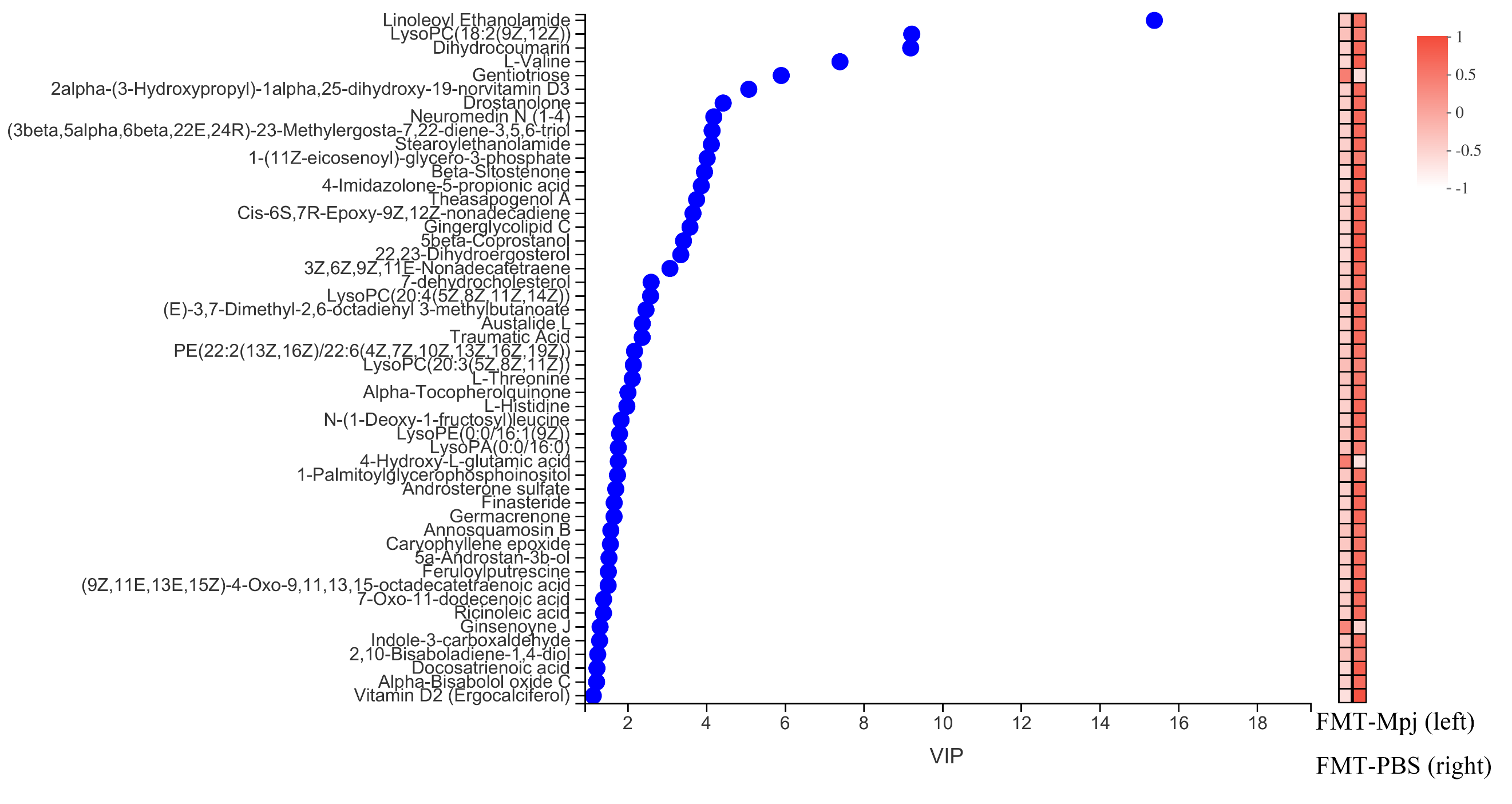


**Supplementary Figure 9.** The VIP bubble plot showed the differential metabolites ordered by the VIP scores and the corresponding expression heatmap between the FMT-Mpj and FMT-PBS**.** The deeper the color is, the higher the level of the metabolite is.


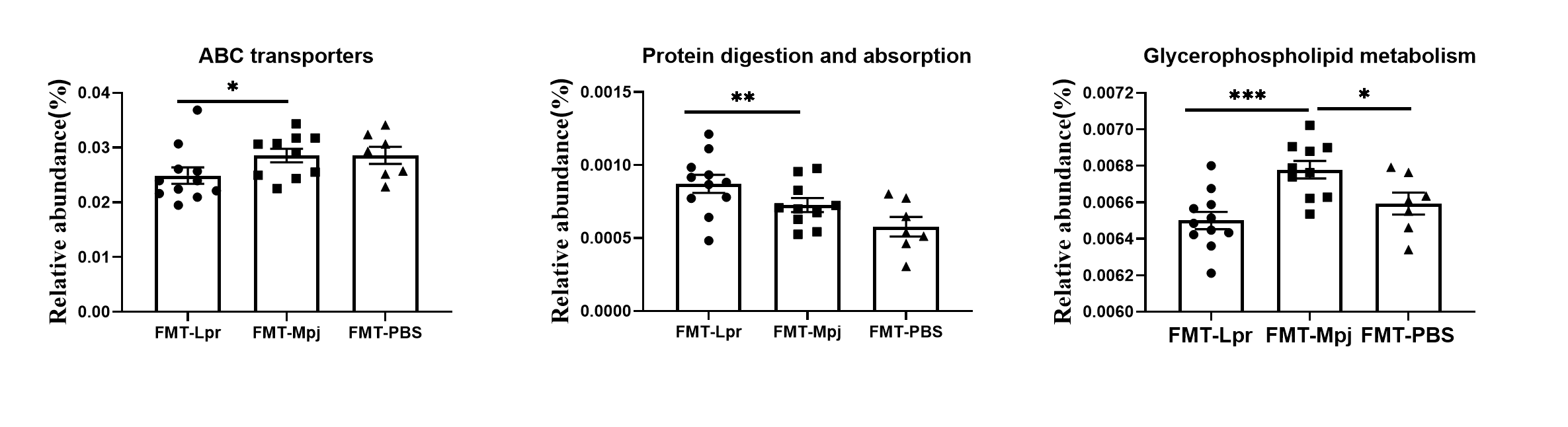


**Supplementary Figure 10.** The relative abundances of the ABC transporters, Protein digestion and absorption, Glycerophospholipid metabolism among the FMT-Lpr, FMT-Mpj and FMT-PBS. *p < 0.05.**p<0.001. FMT-Lpr, n=11; FMT-Mpj, n=10; FMT-PBS, n=7.


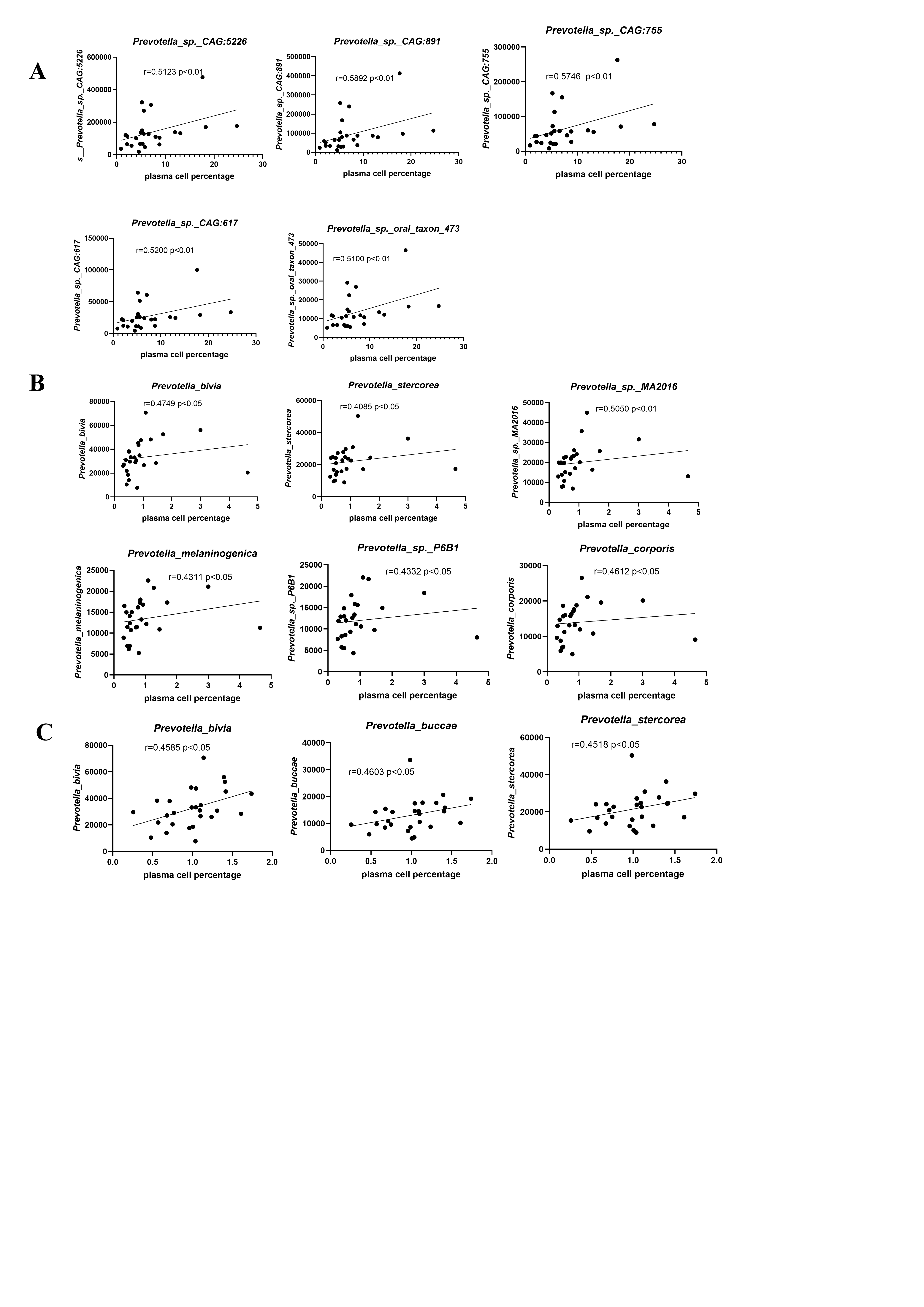


**Supplementary Figure 11.** (A) The association of the abundances of the *prevotella* taxa with the percentage of plasma cells in the large intestine. (B)The association of the abundances of the *prevotella* taxa with the percentage of plasma cells in the peyer’s patches. (C)The association of the abundances of the *prevotella* taxa with the percentage of plasma cells in the mesentery lymph nodes. only r values>0.4 and p values<0.05 were shown.
